# Rapid preparation of binary mixtures of sodium carboxylates as anodes in sodium-ion batteries†

**DOI:** 10.1039/d3ta06928a

**Published:** 2024-04-12

**Authors:** Aamod V. Desai, Romy Ettlinger, Heitor S. Seleghini, Maximillian G. Stanzione, Joel M. Cabañero, Sharon E. Ashbrook, Russell E. Morris, A. Robert Armstrong

**Affiliations:** a EaStCHEM School of Chemistry, University of St Andrews North Haugh St Andrews KY16 9ST UK rem1@st-andrews.ac.uk ara@st-andrews.ac.uk; b The Faraday Institution, Quad One Harwell Science and Innovation Campus Didcot OX11 0RA UK

## Abstract

Sodium-ion batteries are emerging as a sustainable solution to tackle the growing global energy demands. In this context, organic electrode materials complement such technologies as they are composed of earth-abundant elements. As organic anodes, sodium carboxylates exhibit promising applicability in a wide range of molecules. To harness the advantages of individual systems and to minimise their limitations, in this work, an approach to form binary mixtures of sodium carboxylates using one-pot, microwave-assisted synthesis is presented. The target mixtures were synthesised in 30 min with disodium naphthalene-2,6-dicarboxylate (Na-NDC) as a common constituent in all. Both components in all mixtures were shown to participate in the charge storage and had a considerable effect on the performance characteristics, such as specific capacity and working voltage, in half and full cell formats. This approach opens a new avenue for enabling organic materials to be considered as more competitive candidates in sodium-ion batteries and promote their use in other material classes to overcome their limitations.

## Abstract 2

An approach to form binary mixtures of sodium carboxylates using efficient microwave-assisted synthesis, as anodes in sodium-ion batteries is demonstrated.

## Graphical abstract

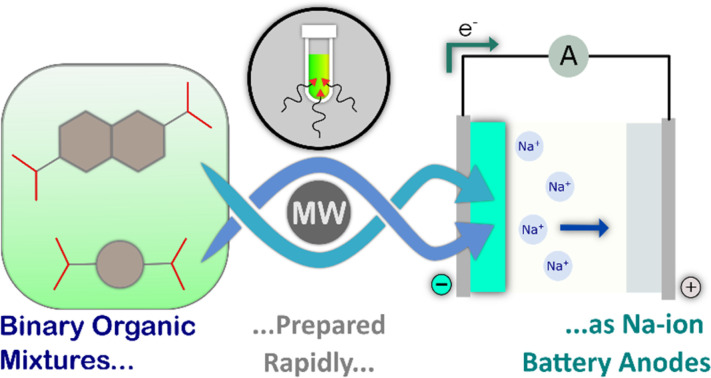

## Introduction

Electrochemical energy storage (EES) devices have gained ever increasing prominence in the pursuit of meeting rising global energy demands.^1^ In this regard, the successful commercialisation of Li-ion batteries (LIBs) has accelerated the development of rechargeable battery technologies.^2^ Owing to the wider abundance of sodium, lower costs of raw materials and safer battery operation, Na-ion batteries (NIBs) have commanded attention as viable candidates to manage energy demands in a more sustainable manner.^3–5^ Although NIBs have a similar working principle to LIBs, a direct transition between electrode materials is seen to be impractical, especially in terms of anodes.^6^ Research into the development and optimisation of anode materials, thus remains central to effective implementation of such technologies.

The field of organic electrode materials (OEMs) in rechargeable batteries has steadily grown over the last decade, strongly linked to the need to foster sustainable materials in battery technologies.^7–9^ These solids are complementary to the sustainability ethos of NIBs, as their precursors can be obtained from biomass or commercial waste,^10,11^ and they are composed of earth-abundant elements.^12^ Furthermore, the synthesis of the active phase is possible using greener and scalable methodologies,^13,14^ and device recycling is environmentally benign.^15^ Owing to the wide possibilities in terms of molecular design, several functional groups are seen to be electrochemically active, such as carbonyl, azo, imine *etc.*^16^ Also, on account of their larger molecular sizes, OEMs in general are better suited for inserting bulkier charge carriers, such as sodium ions.^17^

Sodium carboxylates are a subclass of OEMs in NIBs, that can reversibly insert sodium ions at low, useful potentials and have moderate to high specific capacities.^18^ In terms of their structures, they are typically framework materials, composed of aromatic dicarboxylate linkers that stack in alternate layers separated by Na–O chains.^19,20^ The carboxylate moiety is understood to function as the reaction centre and based on the aromatic backbone and presence of any secondary functional groups, the electrochemical ion-insertion has varied potentials and cycling stabilities.^21,22^ For example, due to its smaller size, disodium terephthalate (Na-BDC) has among the highest capacity in this class of materials,^23,24^ while disodium stilbene-4,4′-dicarboxylate (Na-SDC) exhibits a high rate performance on account of extended π-conjugation.^25^ While some individual compounds may have more favourable properties than others, their performance can be improved only to a limited extent. Combining different components might enable the final material to inherit all benefits and possibly offer a more competitive solution. For example, the use of biphasic materials over single phase solids has shown an improved performance in the area of layered oxide cathode materials.^26–30^ Although unexplored thus far, OEMs can benefit from a similar route, not only to be able to deliver electrochemical properties that meet the demands of the target application, but also to make these materials more competitive relative to the current benchmark solids.

**Fig. 1:**
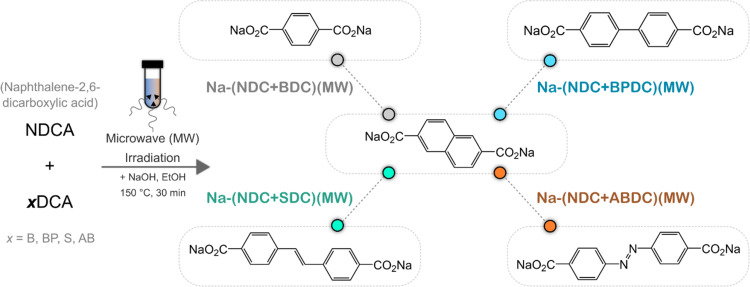
Reaction scheme showing the preparation of different binary mixtures with molecular structures for the basic unit of the sodium carboxylates (BDCA – benzene-1,4-dicarboxylic acid; BPDCA – biphenyl-4,4′-dicarboxylic acid; SDCA – stilbene-4,4′-dicarboxylic acid; ABDCA – azobenzene-4,4′-dicarboxylic acid).

To test this approach in the domain of sodium carboxylates, the rapid preparation of binary mixtures as anode materials in NIBs is presented using a one-pot microwave (MW)-assisted synthesis pathway. The use of MW-irradiation and one-pot reaction aligns with the principles of green chemistry to improve the efficiency of synthesis and minimise waste. Disodium naphthalene-2,6-dicarboxylate – Na-NDC(MW) [referred to as Na-(NDC + NDC)(MW) hereafter when used for comparison], was chosen as one component for every mixture, as its MW-assisted preparation and performance in NIBs was previously optimised.^13^ Binary mixtures were prepared in a 1 : 1 ratio, and to investigate the generality of the approach, four other sodium carboxylates were used, namely, Na-BDC,^23,24^ Na-BPDC,^20^ Na-SDC^25^ and Na-ABDC^31^ [Na-BPDC – disodium biphenyl-4,4′-dicarboxylate; Na-ABDC – disodium azobenzene-4,4′-dicarboxylate] (Fig. 1). Although Na-ABDC has a similar molecular structure to the other sodium carboxylates, the redox activity is driven by the azo bond, at a much higher potential.^31^ The mixtures were characterised using several techniques and their electrochemical performance was examined in both half and full cell formats.

## Experimental

### Materials

The reagents – 2,6-naphthalenedicarboxylic acid (NDCA; 95%, Sigma-Aldrich), terephthalic acid (BDCA; 98%, Sigma-Aldrich), 4,4′-biphenyldicarboxylic acid (BPDCA; 98%, Acros Organics), 4,4′-stilbenedicarboxylic acid (SDCA; 95%, TCI Chemicals), azobenzene-4,4′-dicarboxylic acid (ABDCA; 95%, TCI Chemicals), sodium hydroxide (NaOH; Fisher Scientific), ethanol (EtOH; VWR Chemicals), methanol (MeOH; Fisher Scientific) were obtained commercially and used as received. The chemicals for electrode preparation, namely, Super C65 (Imerys Graphite & Carbon), sodium carboxymethyl cellulose (CMC; degree of substitution 1.2, Sigma-Aldrich) were also obtained from commercial sources.

### MW synthesis of single component sodium carboxylate

The respective dicarboxylic acid (1 mmol) and sodium hydroxide (0.11 g, 2.75 mmol) were added as solid to a reaction tube, followed by addition of EtOH (4 mL). The tube was capped and heated in a microwave reactor (Biotage Initiator) at 150 °C for 30 min, with a pre-heat stirring under ambient conditions for 10 min. A microwave frequency of 2.45 GHz was used to heat the reaction and the reactions were performed at set temperature and time mode. After completion of microwave irradiation, the hot solutions were immediately cooled using pressurised air. Upon cooling, the solid precipitate was filtered off and washed with EtOH (4 × 4 mL) and MeOH (2 × 4 mL). The solid was dried in an oven overnight at 80 °C.

### Solution synthesis of single component sodium carboxylate

The synthesis of Na-ABDC and Na-SDC using solution-based approach was followed using previously reported methods.^25,31^ For preparing Na-ABDC, azobenzene-4,4′-dicarboxylic acid (0.2 g, 0.74 mmol) and NaOH (0.074 g, 1.85 mmol) were added to a flask containing EtOH (15 mL). The reaction mixture was sonicated for 20 min, followed by stirring at room temperature for 24 h. The solid was filtered off, washed with excess EtOH, and dried in an oven at 80 °C overnight. To synthesise Na-SDC, 4,4′-stilbenedicarboxylic acid (1.0 g) and NaOH (0.31 g) were suspended in EtOH (30 mL) at room temperature. The reaction mixture was then allowed to stir overnight at room temperature. The product was collected using filtration and washed with excess EtOH. The solid was dried in an oven at 80 °C before further characterisation.

### MW synthesis of binary mixtures of sodium carboxylate

The same synthesis procedure was employed to prepare binary mixtures as the one for synthesising single components, except each of the carboxylic acids were added 0.5 mmol to the reaction tube. The yields of the products were as follows: Na-(NDC + BDC)(MW) 0.183 g, Na-(NDC + BPDC)(MW) 0.225 g, Na-(NDC + SDC)(MW) 0.244 g, and Na-(NDC + ABDC)(MW) 0.205 g.

### Structural characterisation

Powder X-ray diffraction (PXRD) patterns were recorded on STOE STADIP diffractometer using Mo K_α1_ radiation at room temperature from 1.5–27.5° (2*θ*) in capillary Debye–Scherrer mode. SEM images were collected using a JEOL JSM-IT800 microscope. The powdered samples were placed on copper tape. Raman spectra were recorded on The Renishaw inVia Qontor Raman microscope using a laser excitation of 532 nm.

### Solid-state NMR spectroscopy

Solid-state NMR spectra were recorded on a Bruker Avance III spectrometer equipped with a 14.1 T or a 9.4 T wide bore magnet. All spectra were acquired by packing the powdered samples into 4.0 mm outer diameter ZrO_2_ rotors.

^13^C CP MAS NMR spectra were acquired on a 14.1 T magnet, rotating at a magic angle spinning (MAS) rate of 12.5 kHz, using 3 ms of contact pulse (ramped for ^1^H) for the transfer of magnetisation from ^1^H to ^13^C. During acquisition, SPINAL-64 (ref. 32) ^1^H heteronuclear decoupling was performed with a pulse length of 6.4 ms and radiofrequency field strength of 55.5 kHz. All ^13^C CP MAS NMR spectra are the result of averaging between 96 and 336 transients, with a recycle interval of 10 s.

^23^Na MAS and MQMAS NMR spectra were acquired on a 9.4 T magnet, at a MAS rate of 14 kHz. The spectra were acquired using a π/12 short-flip angle pulse with a radiofrequency field strength of approximately 116 kHz. All the spectra are the result of averaging 512 transients, with a recycle interval of 8 s for all samples. The ^23^Na MQMAS spectrum was acquired using a z-filter pulse sequence,^33^ using a radiofrequency field strength of approximately 116 kHz for the triple-quantum excitation and conversion pulse, while the CT-selective pulse had a strength of approximately 10 kHz. The recycle delay used in the MQMAS spectrum was also 8 s, with 72 transients being acquired for each *t*_1_ point. The MQMAS spectrum is shown after a shearing transformation to allow the projection of the isotropic spectrum directly onto the indirect dimension and is referenced using the convention in Pike *et al.*^34^

^13^C CP MAS spectra are shown referenced relative to tetramethylsilane (TMS) and the ^23^Na MAS ones relative to 1 M NaCl, using l-alanine (CH_3_, 20.5 ppm) and solid NaCl (7.2 ppm) as secondary references.

### Electrochemical characterisation

The working electrodes were prepared by mixing the active material (60 wt%), conductive carbon (Super C65, 30 wt%) and carboxymethyl cellulose (CMC, 10 wt%) as the binder. The slurry was prepared by hand grinding a mixture of the active material and conductive carbon which was added to the binder solution in water and stirred for 3 h. The slurry was cast onto aluminium foil (Advent Research) using a doctor blade and air dried for 2 h. Electrodes were then punched (diameter ∼ 12 mm) and dried overnight in a vacuum oven at 110 °C. The approximate active mass loading for Na-(NDC + BDC)(MW), Na-(NDC + BPDC)(MW), Na-(NDC + SDC)(MW) and Na-(NDC + ABDC)(MW) were 1.78 mg cm^−2^, 1.70 mg cm^−2^, 1.77 mg cm^−2^ and 1.88 mg cm^−2^ respectively.

Both the half and full cell studies were performed on coin cells (CR2032), with glass fiber separator (Whatman GF/F) and electrolyte as NaPF_6_ in ethylene carbonate (EC) and diethyl carbonate (DEC) (1 : 1, v/v) (Kishida Chemical), in an argon-filled glovebox (MBraun) with oxygen and water content < 1 ppm. For half cells sodium metal (Sigma-Aldrich) was used as the counter electrode, while for full cells the cathode material – Na_(0.79±0.05)_Ni_(0.27±0.05)_Mn_(0.42±0.05)_Mg_(0.15±0.05)_Ti_(0.17±0.05)_O_(2±0.05)_ was used on a C-coated Al foil with a composition of 92% active, 3% conducting additive, 5% binder. The galvanostatic cycling studies were performed at 30 °C on a Biologic BCS-805 modular battery testing system and raw data was analysed using BT-Lab software or cycled on a Neware BTS tester and data processed using BTSDA software. Cyclic voltammograms (CV) were recorded at a scan rate of 0.1 mV s^−1^ in the potential window of 0.01–2.5 V. For all half-cell studies, the calculations were based on the mass of the active material, except for a cell involving only carbon and binder, where the calculation was based on the mass of the carbon. In case of full cells, the calculations were based on the mass of the active material in the anode, referred to as AM-anode. Electrochemical impedance spectroscopy (EIS) measurements were recorded on symmetric coin cells which were prepared by disassembling half cells run over 1 cycle between 0.01 and 2.5 V at a current density of 25 mA g^−1^. Fresh cell parts, separator and electrolyte were used for the reassembly and all cells were allowed to rest for 4 h before measurement. EIS spectra were collected at 30 °C on a Biologic BTS-805 battery testing system in the frequency range of 10 kHz to 10 mHz with a voltage amplitude of 10 mV.

## Results and discussion

In the first step of the current study, two individual sodium carboxylates, namely, Na-SDC(MW) and Na-ABDC(MW), were synthesised using MW irradiation using the same protocol previously employed for preparing Na-NDC(MW), Na-BDC(MW) and Na-BPDC(MW).^13^ Powder X-ray diffraction (PXRD) patterns were compared to the materials obtained from solution-based synthesis,^25,31^ validating the successful formation of the respective products (Fig. S1 and S2†). Leading on from this successful synthesis, the preparation of materials with mixed sodium carboxylates in a one-pot approach was attempted by using a NDCA : *x*DCA precursor ratio of 1 : 1. In all the cases the reaction was heated under microwave irradiation for 30 min (details in experimental methods). For all four mixed materials examined in this work, the PXRD patterns indicated the presence of the individual sodium carboxylates with no trace of a crystalline side-product (Fig. S3†). Raman spectra showed that in the case of the mixtures, the predominant signals corresponding to only one component were visible in all the cases (Fig. S4†). The signals in all the mixtures originating from Na-NDC(MW) were either of low intensity or not visible, which indicated one component of the mixture dominated the surface of the particles. This observation was also seen in SEM images, where the bigger crystallites were covered with a layer of particles having one kind of morphology (Fig. S5, S7, S9 and S11†). The surface morphology in the mixtures was similar to that of the respective single component sodium carboxylates (Fig. S6, S8, S10 and S12†). In a previous study,^13^ crystallites of Na-NDC(MW) were shown to have a block shaped morphology, which would indicate the formation of an interparticle growth in the mixtures with Na-NDC(MW) at the core. The utility of employing a one-pot synthesis for preparing binary mixtures could be further highlighted when compared to the SEM images of the materials formed by grinding individual sodium carboxylates (Fig. S13†). The crystallites in the latter case had separate conglomerates of one material.

^23^Na MAS and ^13^C CP MAS NMR spectra were obtained for all binary mixtures and the corresponding single-component sodium carboxylates (Fig. S14–S21†). All spectra of the binary mixtures show no distinguishable change from those expected from the combination of the spectra obtained from the single component sodium carboxylates. This indicates that the synthesis leads to a mixture of compounds with the same short-range structure as exhibited by the individual materials produced from single-component syntheses. As all the ^23^Na MAS spectra were recorded using a short flip angle (*i.e.*, are expected to be quantitative) it was possible to estimate the proportion of each component present in the mixtures, and the values are shown in Table S1,† which vary from 1 : 1 to ∼1 : 2. The deviations from the starting 1 : 1 composition of the starting mixtures could be the result of the higher acidity (and as a consequence the reactivity with NaOH) of the organic acids when compared with NDCA, or perhaps the difference in solubility of each component in methanol and ethanol which were used during the sample washing. Elemental analysis for the binary mixtures confirmed the results estimated from the NMR fitting, with a slightly greater deviation for the mixture containing Na-BDC than the others although still within acceptable limits (Table S2†). When comparing the Na-BDC ^13^C CP MAS NMR spectrum with that from Whewell *et al.*^35^ a second carboxylate resonance at approximately 173 ppm with lower intensity is observed possibly indicating that some of the H-atoms in the carboxylic groups are not fully exchanged for sodium atoms. This idea is also supported by the ^23^Na MAS and MQMAS spectra of Na-BDC (Fig. S18 and S22†) where there is also a low-intensity third sodium site centred at approximately −25 ppm from the sodium atoms close to carboxylic groups in contrast with the two sites from Whewell *et al.*^35^ Such defects should not influence the cycling performance when compared with the fully sodiated Na-BDC, as upon the first charge–discharge cycle the H-atoms will be irreversibly exchanged for Na atoms.^22^ The single carboxylate resonance (∼175 ppm) in the Na-BPDC ^13^C CP MAS spectrum (Fig. S15†) shows that the MW synthesis conditions used result in the production of the anhydrous phase of the Na-BPDC.^20,35^

Working electrodes for all the materials were prepared using a water-soluble binder (10 wt%, carboxymethyl cellulose [CMC]), 60 wt% of active material (synthesised mixtures of sodium carboxylates) and conductive carbon (30 wt%). To gauge an initial response, cyclic voltammetry (CV) and galvanostatic cycling was performed for all mixtures and Na-(NDC)(MW) in half cells with sodium metal as the counter electrode. The reduction peak in the CV for Na-(NDC + NDC)(MW) was seen to stabilise at 0.3 V after the first few cycles with a shoulder at 0.35 V, although the first sodiation peak was observed at 0.17 V (Fig. S23†). The oxidative peaks remained relatively unchanged over the 5 cycles at 0.51 V, which is in good agreement with the previous study.^36^ This redox couple was also evident in all the mixtures at the same potential. The low voltage (<0.1 V) redox couple could be ascribed to insertion of Na-ions in the conductive carbon. The feature around 0.6 V in the first sodiation step, more prominent in the case of mixtures, can be assigned to the formation of the solid-electrolyte interphase (SEI). In the case of Na-(NDC + BDC)(MW), a broad reduction profile was observed with two peaks stabilising at 0.17 V and 0.33 V after the first cycle. In the desodiation scan, a broad feature peaked at 0.53 V. For Na-(NDC + BPDC)(MW) and Na-(NDC + SDC)(MW), similar characteristics of a single reduction peak and two distinct oxidative peaks were observed. Overlapping of peaks from the 2^nd^ cycle onwards for Na-(NDC + BPDC)(MW) indicated better reversibility, and the oxidation peak at 0.65 V further validated the formation of fully sodiated Na-BPDC phase in the synthesised mixture.^20^ Similar to the previous report, an additional peak at 0.77 V was observed in the first few cycles for Na-(NDC + SDC)(MW), which decreased in the following cycles with increasing intensity for the peak at 0.65 V corresponding to the oxidation of sodiated Na-SDC.^25^ Two independent redox couples were seen in the case of Na-(NDC + ABDC)(MW). At higher potential, two sharp oxidation peaks were observed at 1.38 and 1.46 V, with only one peak for sodiation at 1.14 V. These values closely relate to the previous work on the single component Na-ABDC.^31^

**Fig. 2:**
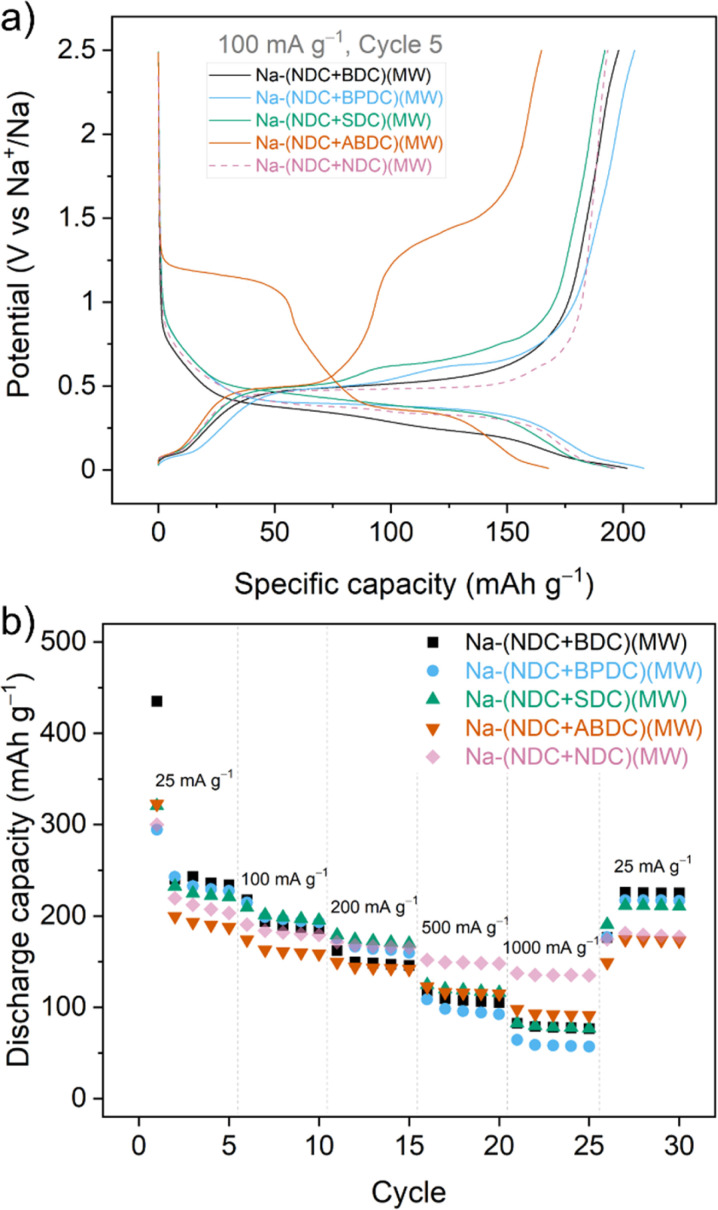
(a) Charge/discharge traces for the 5^th^ cycle for all the synthesised mixtures upon galvanostatic cycling at 100 mA g^−1^ between 0.01 and 2.5 V. (b) Rate performance when cycled five times at each current rate.

Galvanostatic cycling was performed at a current rate of 100 mA g^−1^. For all the synthesised mixtures, a two-step insertion and de-insertion process was evident from the voltage traces, which validated participation by both components in all the mixtures (Fig. 2a). Differential capacity plots further indicated these steps, with slightly broadened peaks in cases of overlapping potentials of individual parts (Fig. S24†). For example, a single, broad oxidation peak was observed for Na-(NDC + BDC)(MW) even though two discernible peaks were observed for the reduction at 0.22 and 0.37 V for Na-BDC^24^ and Na-NDC,^36,37^ respectively (Fig. S24†). Owing to a considerable difference in the working potentials for the individual components, well separated peaks were seen in the differential capacity profile for the Na-(NDC + ABDC)(MW). In terms of specific capacities, all the mixtures showed an expected pattern of lower capacity with increasing molecular size of the second component (Fig. S25†). In addition, the mixtures had a noticeable difference in the cycling capacities compared to Na-(NDC + NDC)(MW). The mixture Na-(NDC + BDC)(MW) exhibited the highest first discharge capacity (385 mA h g^−1^), with significant irreversibility upon desodiation. This can partly be ascribed to the formation of SEI and sodium-ion insertion into the conductive additive (Fig. S26†).^38^ Also, the loss of reversible capacity in the first sodiation could be attributed to the presence of defects in Na-BDC. From the second cycle onward, the discharge capacity remained stable over 100 cycles. Na-(NDC + BPDC)(MW) demonstrated slightly higher discharge capacities over Na-(NDC + BDC)(MW) for the first few cycles, with better reversibility. Na-(NDC + SDC)(MW) showed one of the highest initial coulombic efficiency (ICE), but the cycling stability was relatively poor. While Na-(NDC + ABDC)(MW) had the lowest specific capacities among the mixtures, it exhibited stable cycling with marginally lower performance compared to the unmixed solid Na-(NDC + NDC)(MW). In terms of initial coulombic efficiency (ICE), Na-(NDC + BDC)(MW) had the lowest value (∼50%) among all mixtures (Fig. S27†). This value for the bicomponent solid was significantly lower than ICE values seen for either of the individual materials (Fig. S28 and S29†). A similar observation was noted for Na-(NDC + BPDC)(MW), although the difference between the ICE values for the mixture and individual compounds was less apparent. For the other two mixtures, the bicomponent materials had ICE values between those for respective individual components. To compare with the single component sodium carboxylates, the discharge capacities are shown in Fig. S30.† Among each of the materials, apart from Na-NDC(MW), Na-BPDC(MW) exhibited the highest ICE value (Fig. S28†) and the most stable cycling performance (Fig. S30†). The above-described pattern in ICE values for the mixtures was retained when the mixtures were cycled at a lower current rate (25 mA g^−1^) (Fig. S31†), suggesting that the trend can not only be correlated to the proximity of the bicomponent crystallites caused by the one-pot reaction, but also to the chemical characteristic of the individual materials in the mixtures. At this current rate (25 mA g^−1^), the specific capacities followed the expected pattern based on the mass of the molecules, with Na-(NDC + BDC)(MW) showing a high discharge capacity of 254 mA h g^−1^ for the 2^nd^ cycle (Fig. S32†). It is worth noting, that these values of specific capacities, cycling stability and ICE for the mixtures are among high performing organic anode materials for NIBs.^39–41^

To further probe the sodium storage in the binary mixtures, rate performance tests were carried out from 25 mA g^−1^ up to a current density of 1 A g^−1^ (Fig. 2b). It is worth highlighting that all the mixtures were able to recover most of their specific capacity when the current was lowered back to 25 mA g^−1^. Apart from at high current (500 mA g^−1^ and 1000 mA g^−1^) the mixtures, other than Na-(NDC + ABDC)(MW), fared better than Na-(NDC + NDC)(MW). The separation at higher current densities could be ascribed to the microstructural differences for single components relative to the mixtures, which can limit ion diffusion. Interestingly, among mixtures the drop in specific capacity was lowest for Na-(NDC + ABDC)(MW) followed by Na-(NDC + SDC)(MW), while Na-(NDC + BPDC)(MW) had a noticeable drop in capacity at higher current densities. To understand the differences in the behaviour of mixtures, electrochemical impedance spectroscopy (EIS) was performed in the symmetric cell format^42,43^ for all the mixtures and single component Na-NDC(MW) after one complete cycle (Fig. S33†). The Nyquist plots for all materials included a depressed semicircle at high-frequency and a sloping line at lower frequencies. The interfacial resistance for all the mixtures was comparatively higher than Na-NDC(MW), which could be linked to the drop in performance at higher current densities in the rate capability tests. Among the mixtures, Na-(NDC + ABDC)(MW) had highest interfacial resistance, while Na-(NDC + BPDC)(MW) exhibited the lowest. The differences in the cell impedance in mixtures may not only be ascribed to the heterogeneity of the electrode, but also to the characteristics of the component other than Na-NDC. In general, molecules with extended conjugation are expected to show better rate performance.^25^ The observation from the mixtures suggests that wider separation between reaction potentials may also contribute to improving the retention of capacities at varying current rates.

**Fig. 3:**
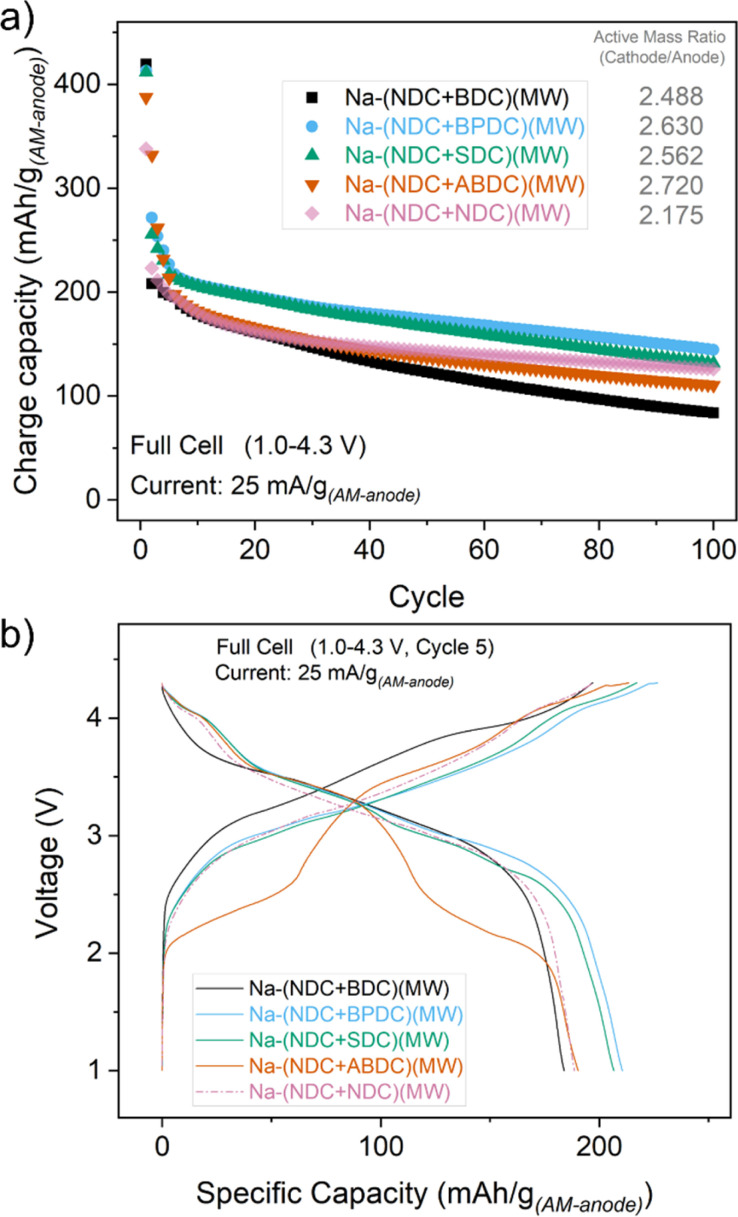
(a) Charge capacity (in terms of anode mass) for all the mixtures in full cells when cycled between 1.0 and 4.3 V at a current density of 25 mA g_(AM-anode)_^−1^. (b) Voltage traces for the 5^th^ cycle.

Encouraged by the half-cell cycling results, cycling studies of all mixtures in full cell formats were completed. Full cells using a layered oxide cathode material were cycled between 1.0 and 4.3 V over 100 cycles at a current rate of 25 mA g^−1^ (with respect to the anode) (Fig. 3a). For reference, the half-cell cycling for the cathode material is shown in Fig. S34.† The mass ratio (cathode/anode) in full cells for the mixtures was maintained between 2.2 and 2.7. The voltage traces in full cells for all mixtures were similar but exhibited subtle differences (Fig. 3b), with the exception of Na-(NDC + ABDC)(MW) which showed two discernible steps. Although Na-(NDC + BDC)(MW) had the highest capacity upon the first charge, it suffered a large loss of reversible capacity in the subsequent cycles. This seemed to have a consequent bearing on the cycling stability and specific capacities and could be linked to the poorer ICE values seen in half cell studies. The materials which had better ICE values in the half cells, Na-(NDC + BPDC)(MW) and Na-(NDC + SDC)(MW), delivered higher, stable capacities with relatively less fade over 100 cycles in full cells. Na-(NDC + ABDC)(MW) exhibited a stable performance after the first few cycles with specific capacities marginally lower than the best performing solids. This pattern was reflected in the coulombic efficiencies (CE) for the full cells, with Na-(NDC + BDC)(MW) having the lowest values of all the materials (Fig. S35†). While the unmixed Na-(NDC)(MW) exhibited better reversibility at low cycle number than the mixtures, both Na-(NDC + BPDC)(MW) and Na-(NDC + SDC)(MW) had higher CE values over extended cycling. For Na-(NDC + ABDC)(MW) the reversibility increased gradually through the first few cycles, and remained stable over the subsequent cycles. Interestingly, when the midpoint voltages (MPV) of the discharge curves for all materials were compared (Fig. S36†), Na-(NDC + ABDC)(MW) mixture fared better than both Na-(NDC + BPDC)(MW) and Na-(NDC + SDC)(MW). In this regard, Na-(NDC + BDC)(MW) performed better with MPV values in excess of 3.3 V over 100 cycles, which were relatively higher than for the unmixed Na-(NDC + NDC)(MW).

These results, when viewed with those for half cells, further highlight the effect of using mixtures. The proximity of individual crystallites can be beneficial in combining specific advantages and maximising the efficiency of charge storage from both materials owing to their well separated reaction potentials. Na-ABDC has a stable cycling performance and a different ion-insertion mechanism compared to the other sodium carboxylates.^31^ A major concern as a standalone anode material, however, is the working voltage. The current approach for forming its mixture, such as with Na-NDC, provides an opportunity to utilise its advantages of smoother rate performance and cycling stability, alongside the observation of a competitive effective working potential. Likewise, the combination of Na-(NDC + BDC)(MW) enables improved achievable specific capacities and working voltages for an electrode containing Na-NDC.

## Conclusions

In summary, the rapid MW-assisted synthesis of four binary mixtures of sodium carboxylates has been demonstrated with Na-NDC(MW) as the common component. The successful preparation of mixtures in one-pot helps align with the principles of green chemistry by reducing waste and obtaining the products in an efficient manner. Both compounds in all mixtures are electrochemically active, despite their different sizes and redox chemistries, and have a combined influence on the specific capacities, ICE values, effective working voltages with respect to the unmixed Na-NDC(MW) and between mixtures. Although the mixtures do not present a linear effect in terms of different electrochemical properties, the reversibility in the initial cycles and the separation of reaction potentials for individual parts were seen to affect the cycling stability and rate performance of the mixtures. The successful demonstration of the approach of using mixtures can facilitate organic electrode materials to overcome their individual limitations and provide a route for them to perform more competitively, and likewise enable the improved performance of active solids in other classes of materials.

## Data availability

The research data underpinning this publication can be accessed at https://doi.org/10.17630/56249b4c-12e8-4b52-8f7d-f792c9b8b791.

## Conflicts of interest

There are no conflicts to declare.

## Supplementary Material

TA-012-D3TA06928A-s001
