# Supplementary material for: Rapid preparation of binary mixtures of sodium carboxylates as anodes in sodium-ion batteries
Source: J Mater Chem A Mater. 2024 Apr 12;12(20):12119–25. doi: 10.1039/d3ta06928a (PMC11107158; doi:10.1039/d3ta06928a)
Supplement: TA-012-D3TA06928A-s001 [file TA-012-D3TA06928A-s001.pdf]

Electronic Supplementary Material (ESI) for Journal of Materials Chemistry A.
This journal is © The Royal Society of Chemistry 2024

Supporting Information

Rapid Preparation of Binary Mixtures of Sodium Carboxylates as Anodes in Sodium-Ion Batteries

Aamod V. Desai,[a,b] Romy Ettlinger,[a] Heitor S. Seleghini,[a] Maximillian G. Stanzione,[a] Joel M. Cabañero Jr.,[a,b] Sharon E. Ashbrook,[a] Russell E. Morris[a,b] and A. Robert Armstrong*[a,b]*

a - EaStCHEM School of Chemistry, University of St Andrews, North Haugh, St Andrews KY16 9ST, United Kingdom.

b - The Faraday Institution, Quad One, Harwell Science and Innovation Campus, Didcot, OX11 0RA, United Kingdom.

Email: rem1@st-andrews.ac.uk ; ara@st-andrews.ac.uk

Figures

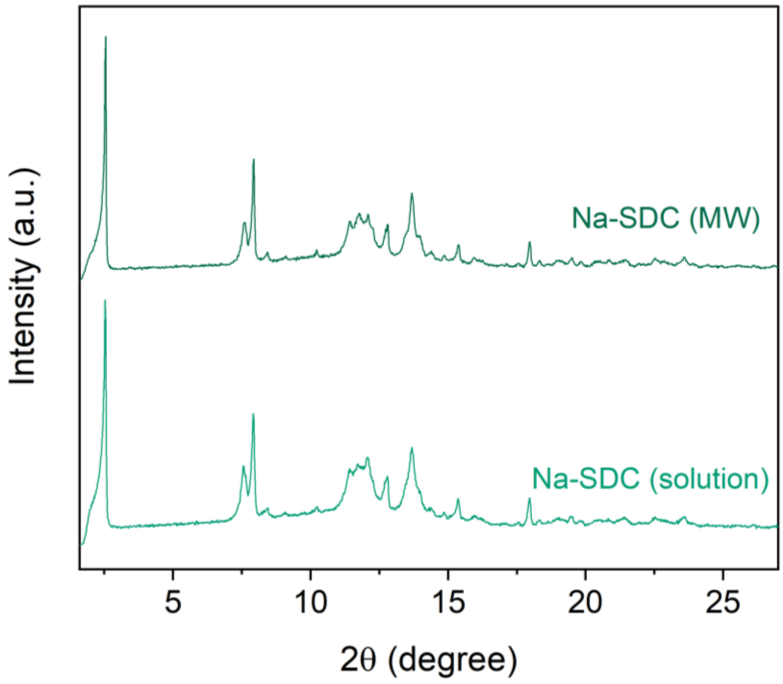

Figure S1. PXRD pattern for Na-(SDC)(MW), compared to the material prepared using a solution-based reaction.

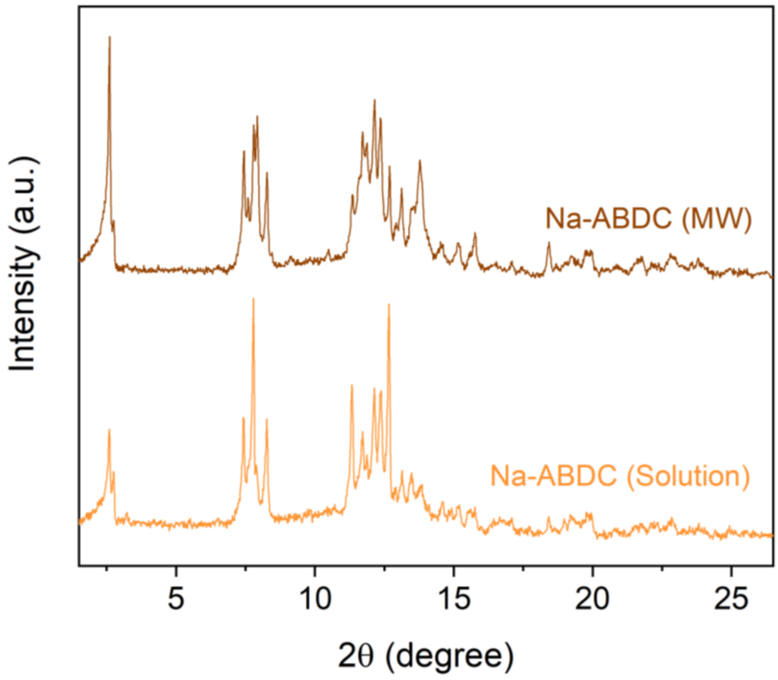

Figure S2. PXRD pattern for Na-(ABDC)(MW), compared to the material prepared using a solution-based reaction.

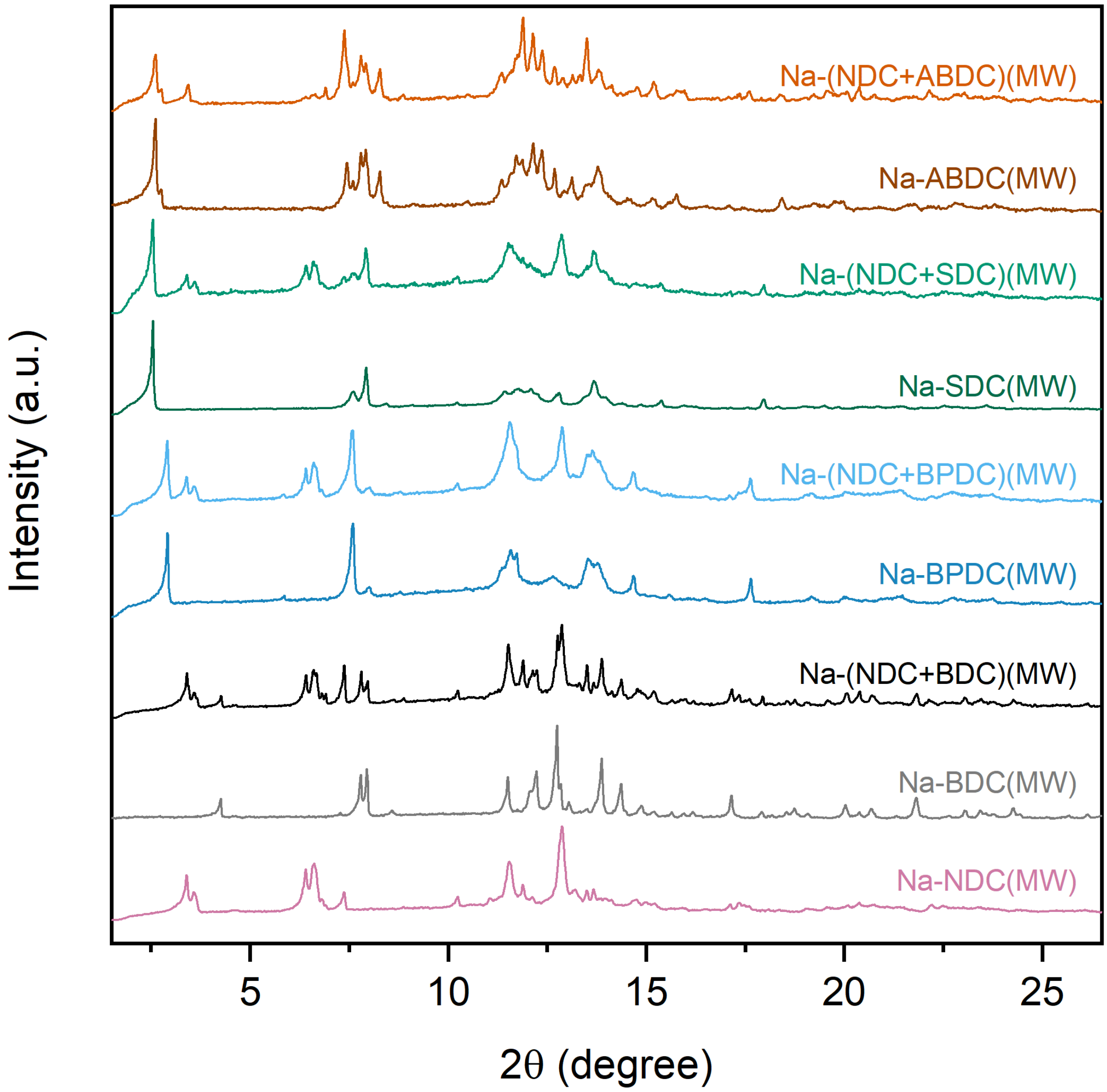

Figure S3. PXRD patterns for all the mixtures and single component sodium carboxylates.

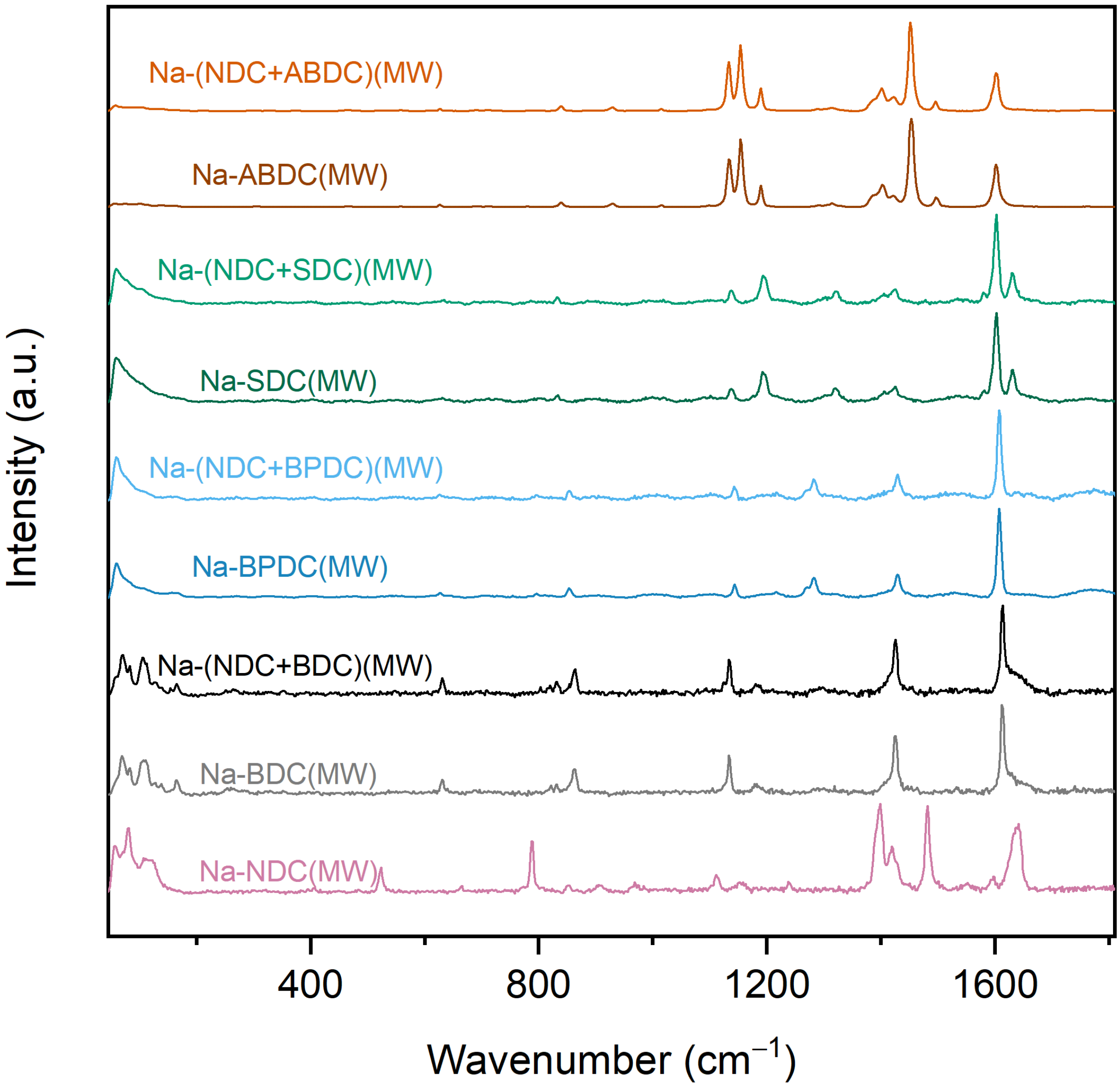

Figure S4. Raman spectra for all the single component sodium carboxylates and binary mixtures.

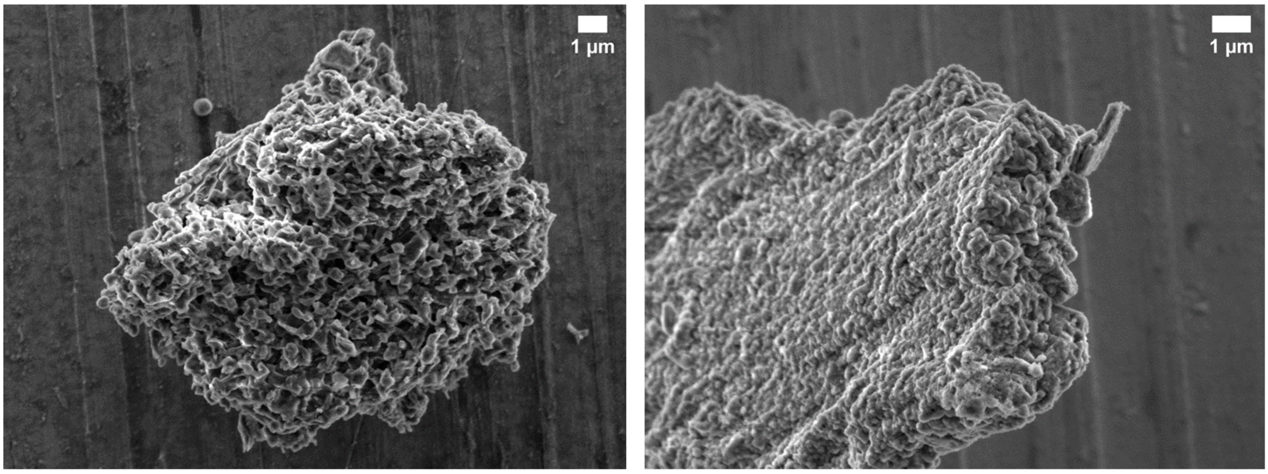

Figure S5. SEM images for as-synthesised powder of Na-(NDC+BDC)(MW).

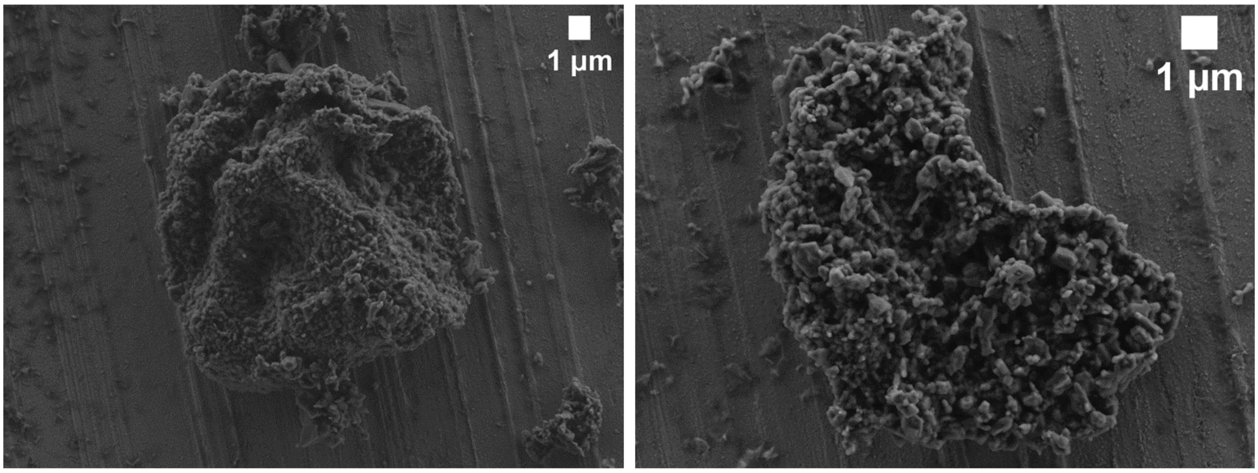

Figure S6. SEM images for as-synthesised powder of Na-BDC(MW).

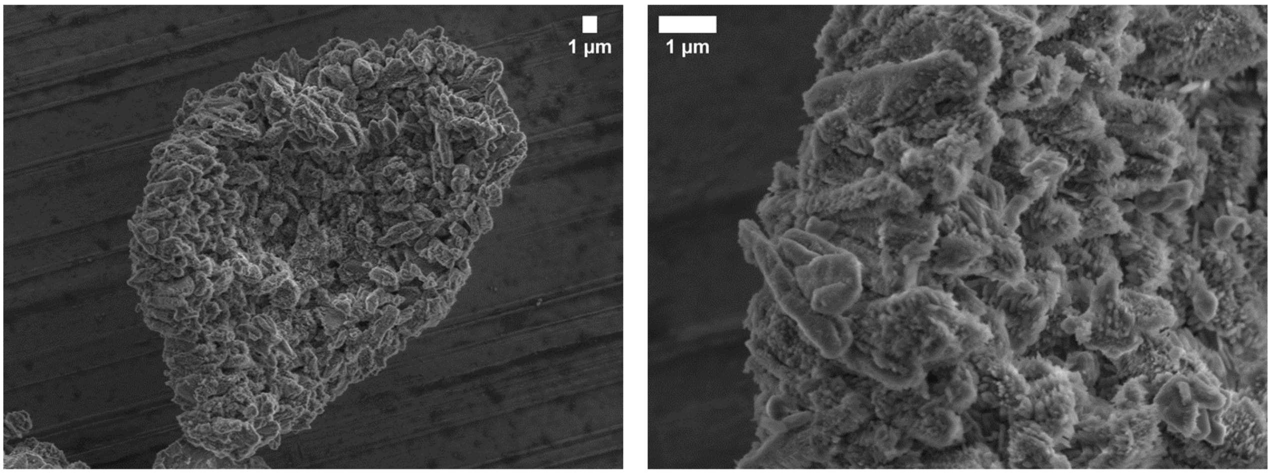

Figure S7. SEM images for as-synthesised powder of Na-(NDC+BPDC)(MW).

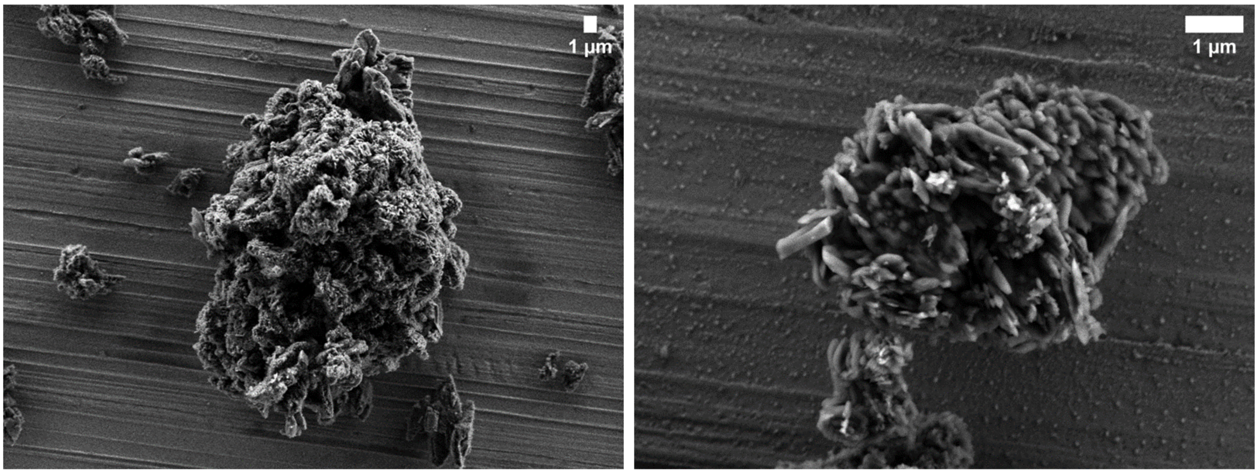

Figure S8. SEM images for as-synthesised powder of Na-BPDC(MW).

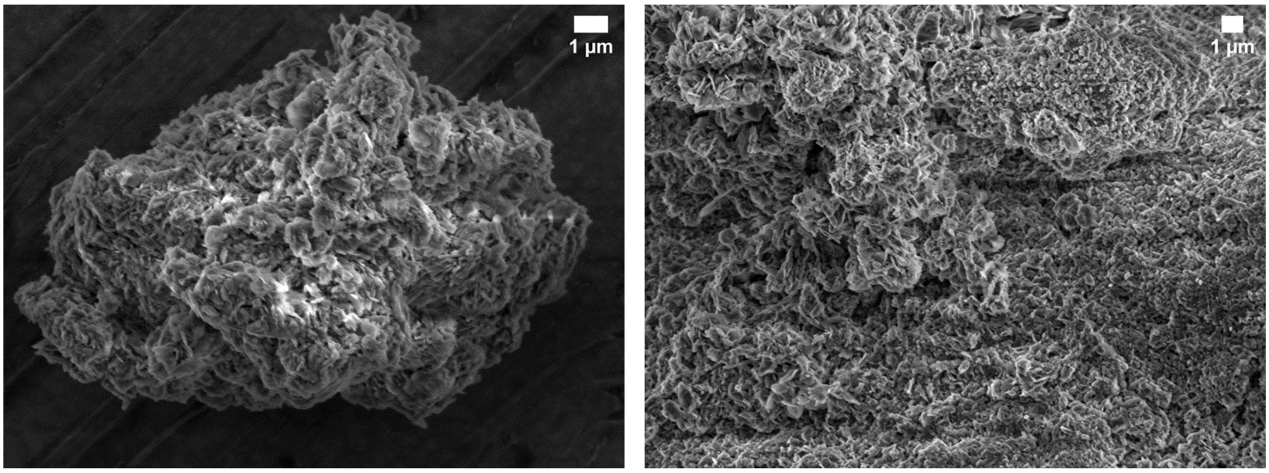

Figure S9. SEM images for as-synthesised powder of Na-(NDC+SDC)(MW).

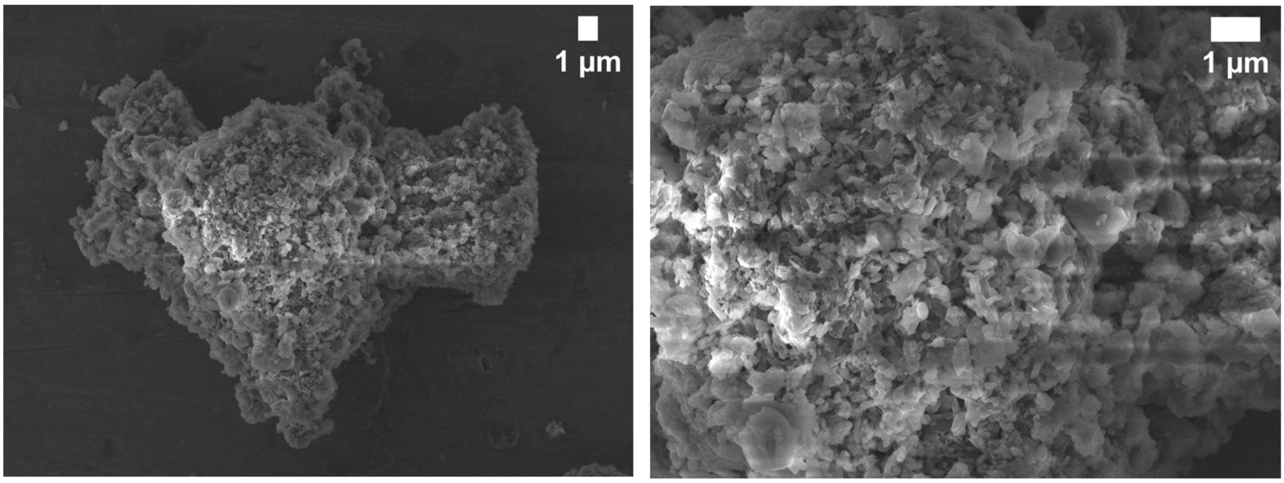

Figure S10. SEM images for as-synthesised powder of Na-SDC(MW).

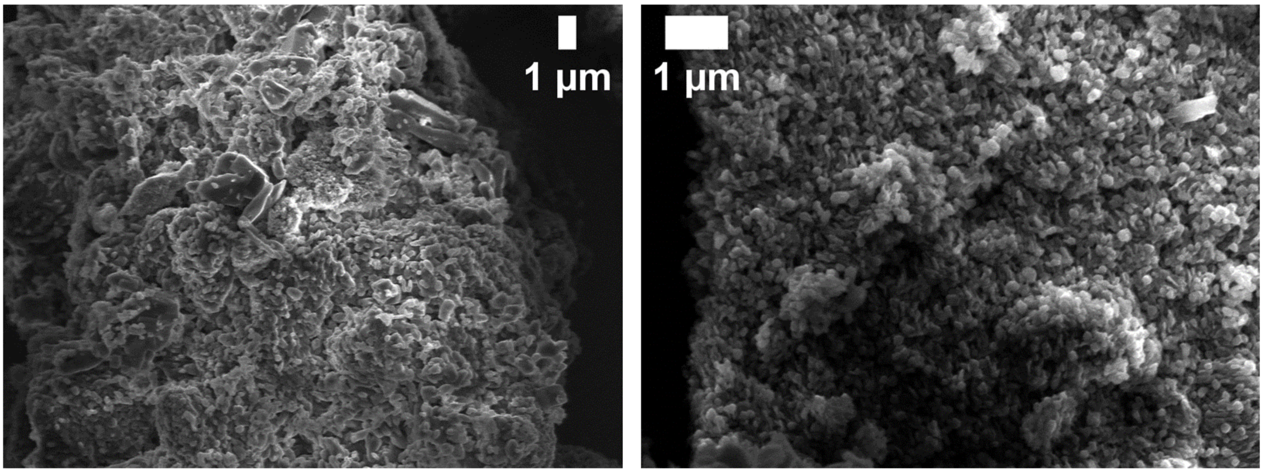

Figure S11. SEM images for as-synthesised powder of Na-(NDC+ABDC)(MW).

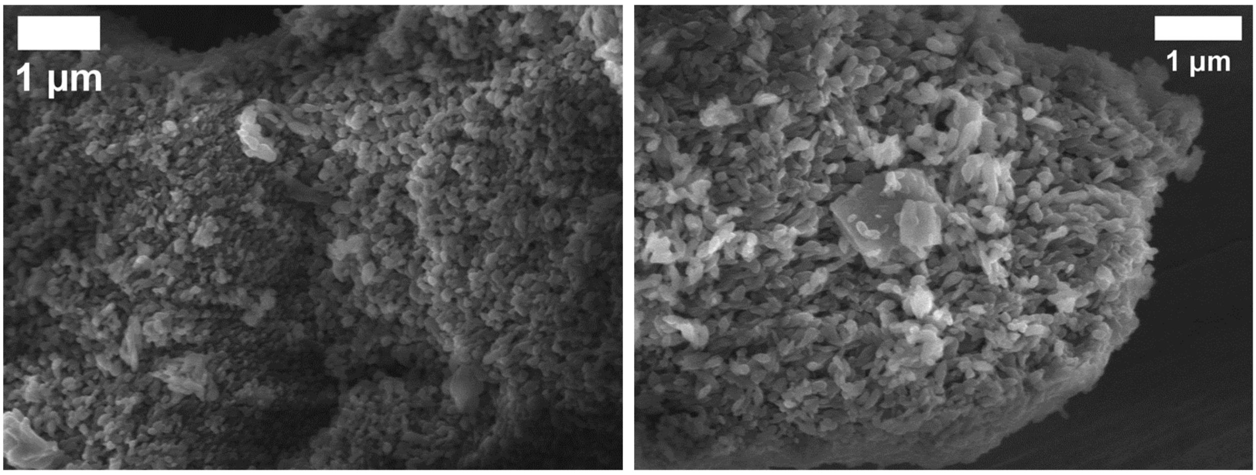

Figure S12. SEM images for as-synthesised powder of Na-ABDC(MW).

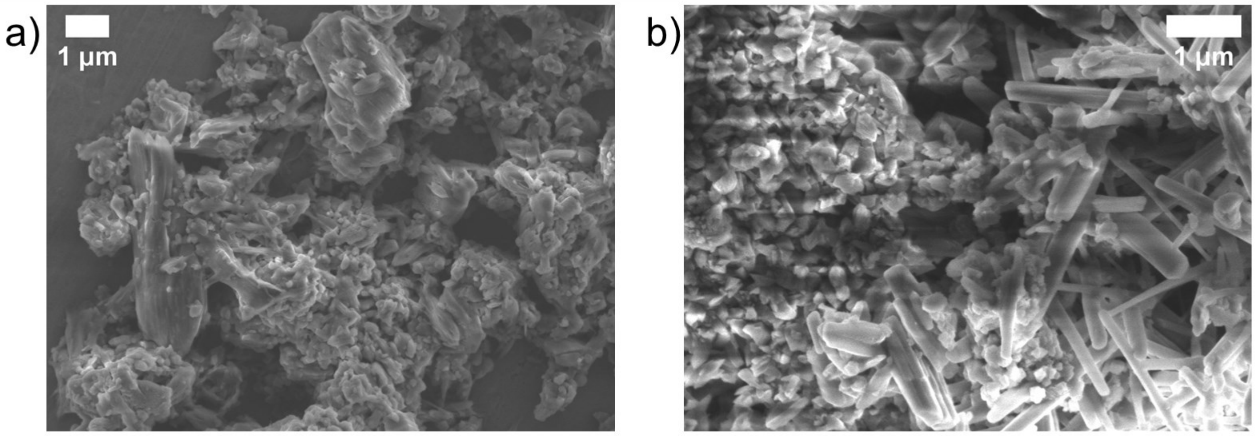

Figure S13. SEM images for mixtures formed by physically mixing individual sodium carboxylates, (a) Na-NDC(MW)+Na-SDC(MW) and (b) Na-NDC(MW)+Na-ABDC(MW).

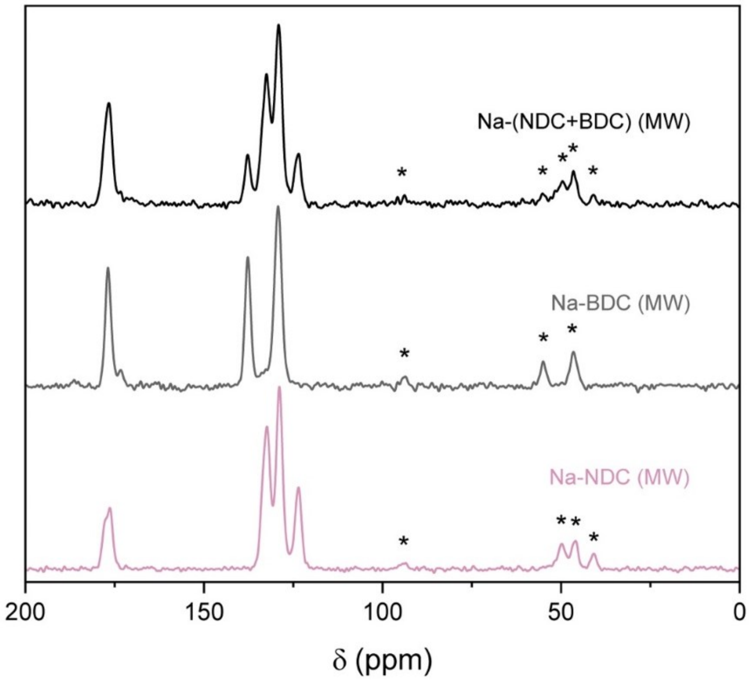

Figure S14. ^{13}C (14.1 T, 12.5 kHz) CP MAS NMR spectra of single component sodium carboxylates [Na-NDC (MW), Na-BDC (MW)] and the mixture Na-(NDC+BDC) (MW). The * symbols indicate spinning sidebands.

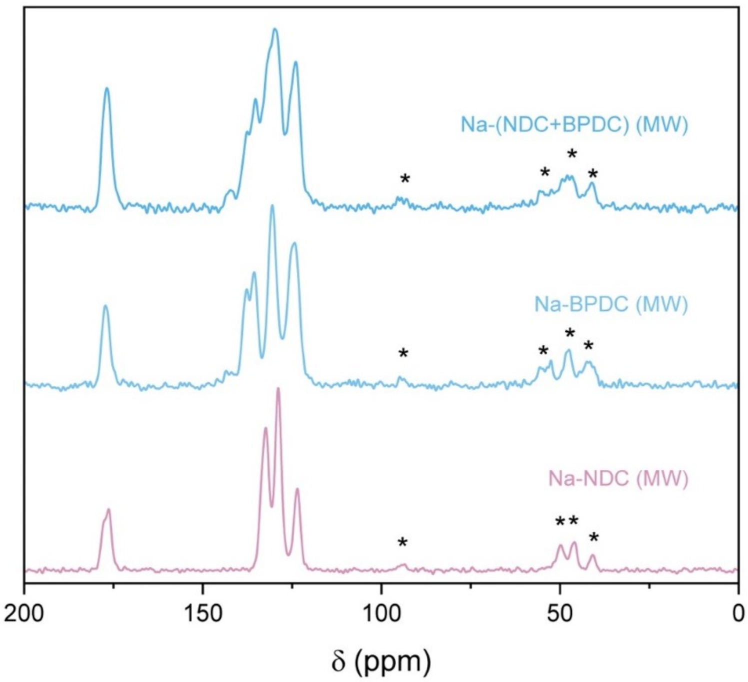

Figure S15. ^{13}C (14.1 T, 12.5 kHz) CP MAS NMR spectra of single component sodium carboxylates [Na-NDC (MW), Na-BPDC (MW)] and the mixture Na-(NDC+BPDC) (MW). The * symbols indicate spinning sidebands.

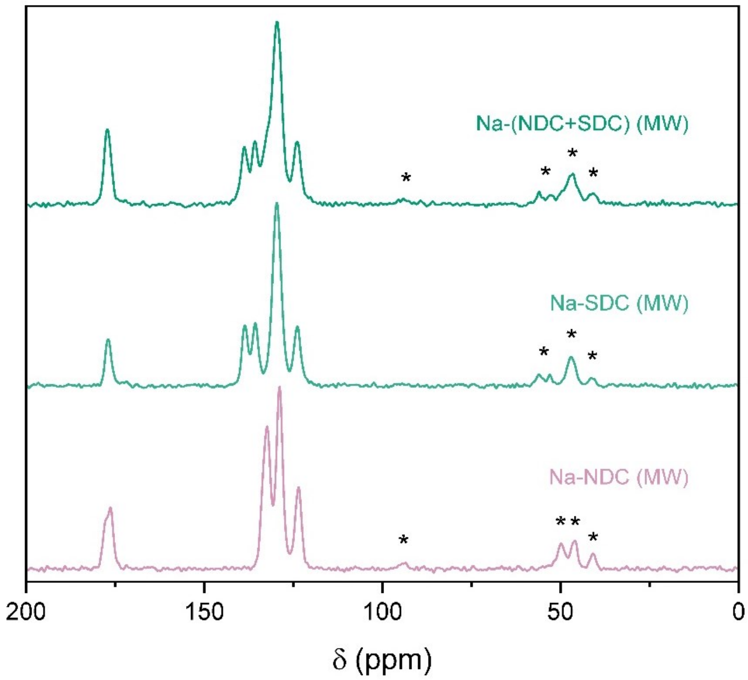

Figure S16. ^{13}C (14.1 T, 12.5 kHz) CP MAS NMR spectra of single component sodium carboxylates [Na-NDC (MW), Na-SDC (MW)] and the mixture Na-(NDC+SDC) (MW). The * symbols indicate spinning sidebands.

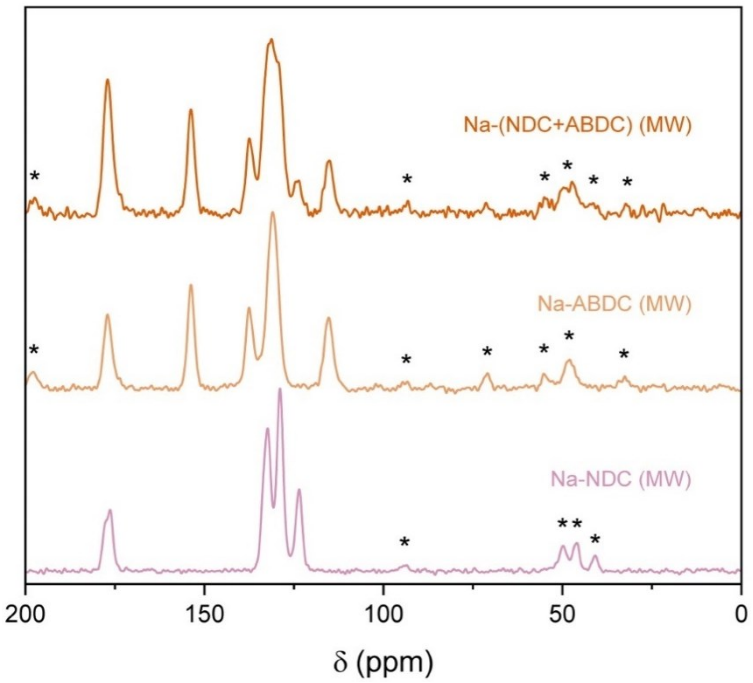

Figure S17. ^{13}C (14.1 T, 12.5 kHz) CP MAS NMR spectra of single component sodium carboxylates [Na-NDC (MW), Na-ABDC (MW)] and the mixture Na-(NDC+ABDC) (MW). The * symbols indicate spinning sidebands.

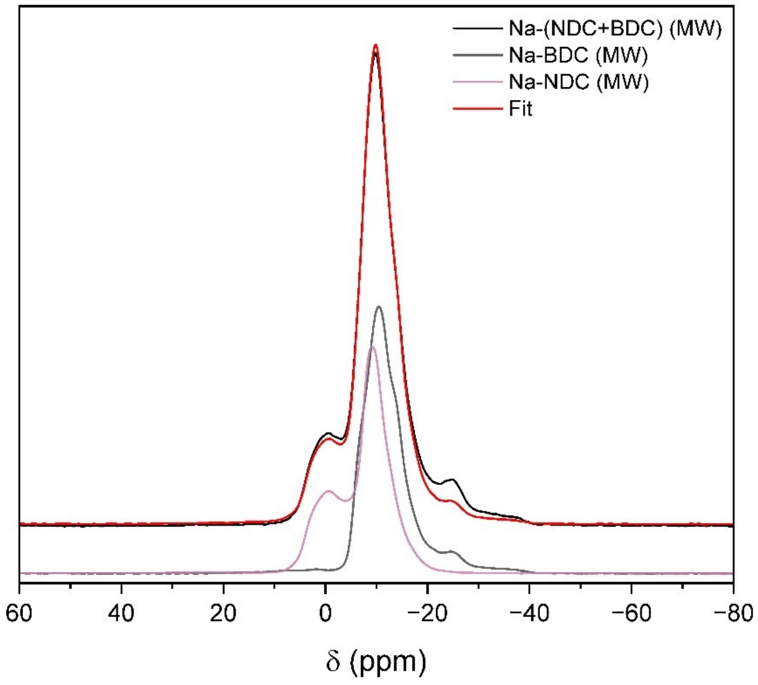

Figure S18. ^{23}Na (9.4 T, 14 kHz) MAS NMR spectra of single component sodium carboxylates [Na-NDC (MW), Na-BDC (MW)] and the mixture Na-(NDC+BDC) (MW).

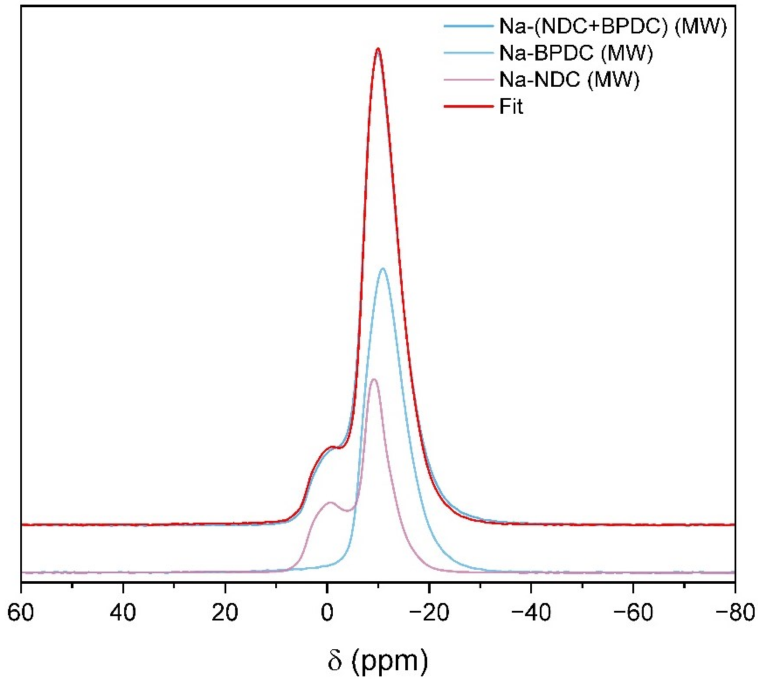

Figure S19. ^{23}Na (9.4 T, 14 kHz) MAS NMR spectra of single component sodium carboxylates [Na-NDC (MW), Na-BPDC (MW)] and the mixture Na-(NDC+BPDC) (MW).

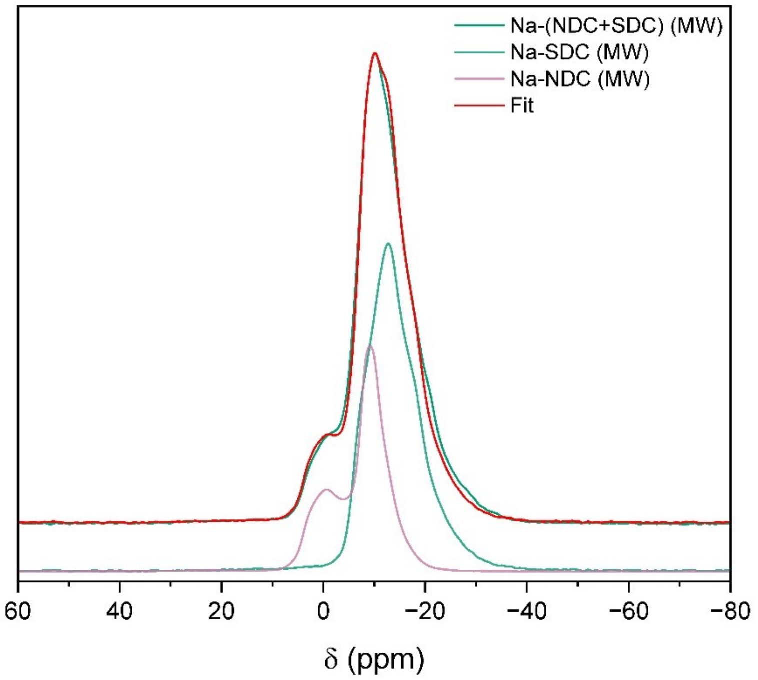

Figure S20. ^{23}Na (9.4 T, 14 kHz) MAS NMR spectra of single component sodium carboxylates [Na-NDC (MW), Na-SDC (MW)] and the mixture Na-(NDC+SDC) (MW).

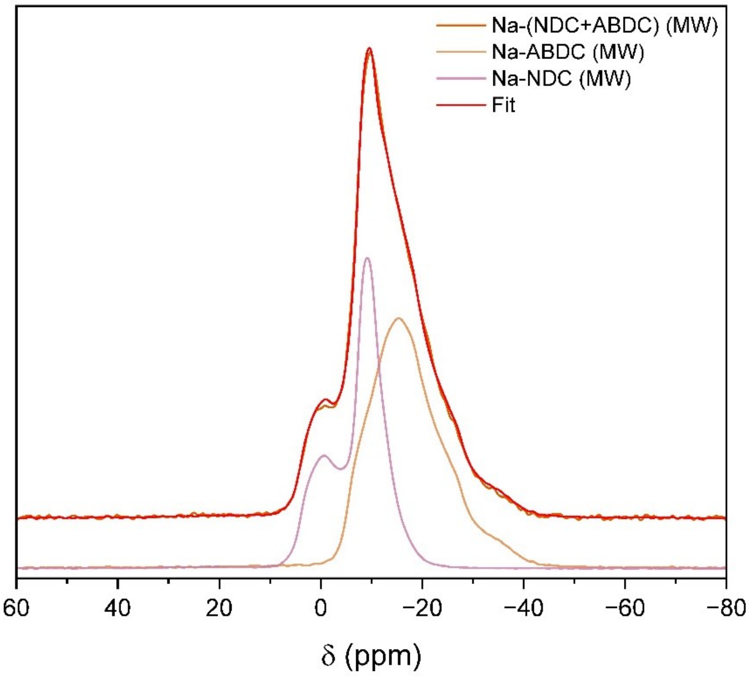

Figure S21. ^{23}Na (9.4 T, 14 kHz) MAS NMR spectra of single component sodium carboxylates [Na-NDC (MW), Na-ABDC (MW)] and the mixture Na-(NDC+ABDC) (MW).

Table S1. Estimation of the molar percentage of each component in the mixtures obtained from the ^{23}Na MAS NMR spectra in Figures S18-S21.

**Mixture**	**Percentage of Na_2NDC**	**Percentage of the other component**
**Na-(NDC+BDC)**	52(3)%	48(3)%
**Na-(NDC+BPDC)**	39(3)%	61(3)%
**Na-(NDC+SDC)**	35(3)%	65(3)%
**Na-(NDC+ABDC)**	41(3)%	59(3)%

Table S2. Elemental analysis results for all the mixtures and corresponding estimated values

**Material**	**Estimated (%) for 1:1 product**	**Found (%) [C, ±0.23; H, ±0.07; N, ±0.09]**	**Estimated (%) for proportions estimated by NMR fitting**
**Na-(NDC+BDC)(MW)**	C, 51.08; H, 2.14	C, 49.92; H, 2.62	C, 51.27; H, 2.15
**Na-(NDC+BPDC)(MW)**	C, 57.16; H, 2.58	C, 55.71; H, 2.72	C, 57.52; H, 2.64
**Na-(NDC+SDC)(MW)**	C, 58.76; H, 2.82	C, 59.04; H, 3.09	C, 59.65; H, 2.95
**Na-(NDC+ABDC)(MW)**	C, 54.37; H, 2.46; N, 4.88	C, 55.27; H, 2.38; N, 5.75	C, 54.21; H, 2.48; N, 5.66

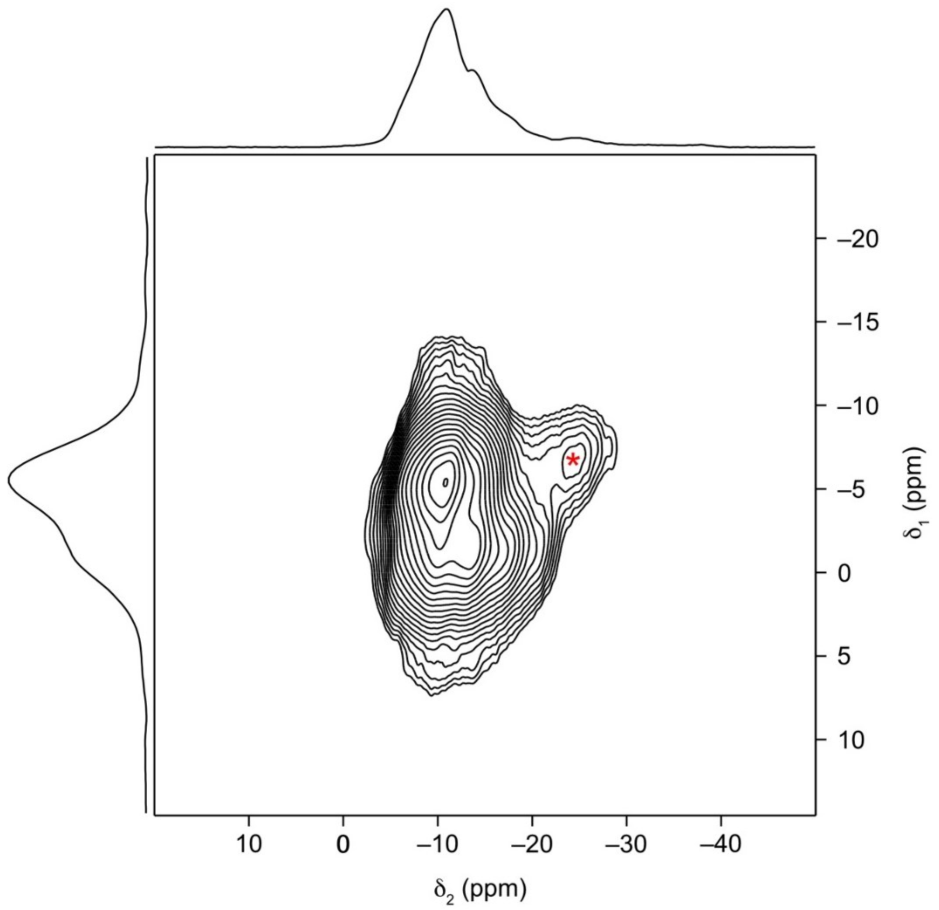

Figure S22. ^{23}Na (9.4 T, 14 kHz) MQMAS NMR spectrum of the single component sodium benzene-1,4-dicarboxylate [Na-BDC (MW)]. The * symbol indicates the additional signal when compared with the Na-BDC spectrum from Whewell *et al.*[1]

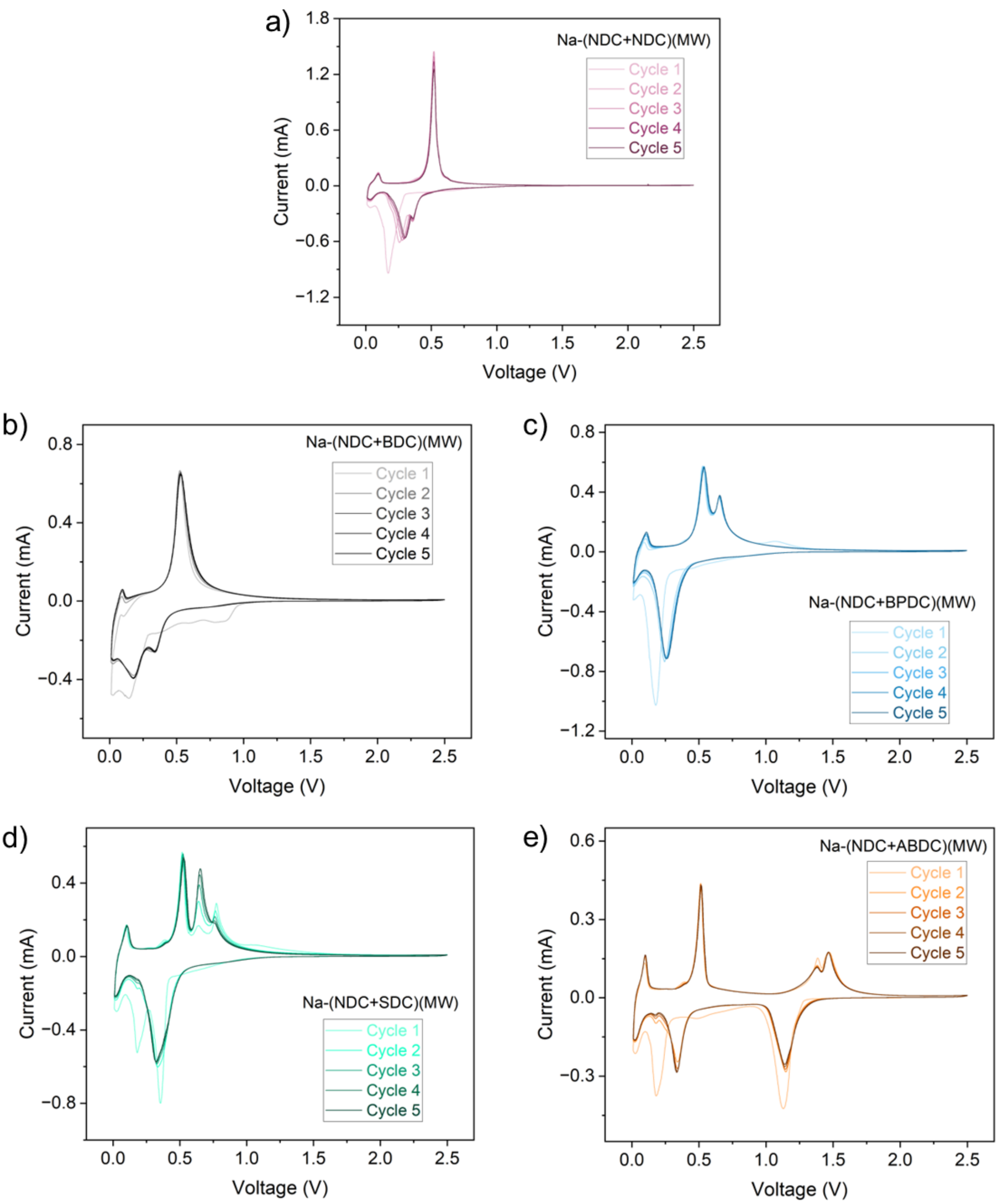

Figure S23. Cyclic voltammograms measured at a scan rate of 0.1 mV s^{-1} for 5 cycles for a) Na-(NDC+NDC)(MW), b) Na-(NDC+BDC)(MW), c) Na-(NDC+BPDC)(MW), d) Na-(NDC+SDC)(MW), e) Na-(NDC+ABDC)(MW).

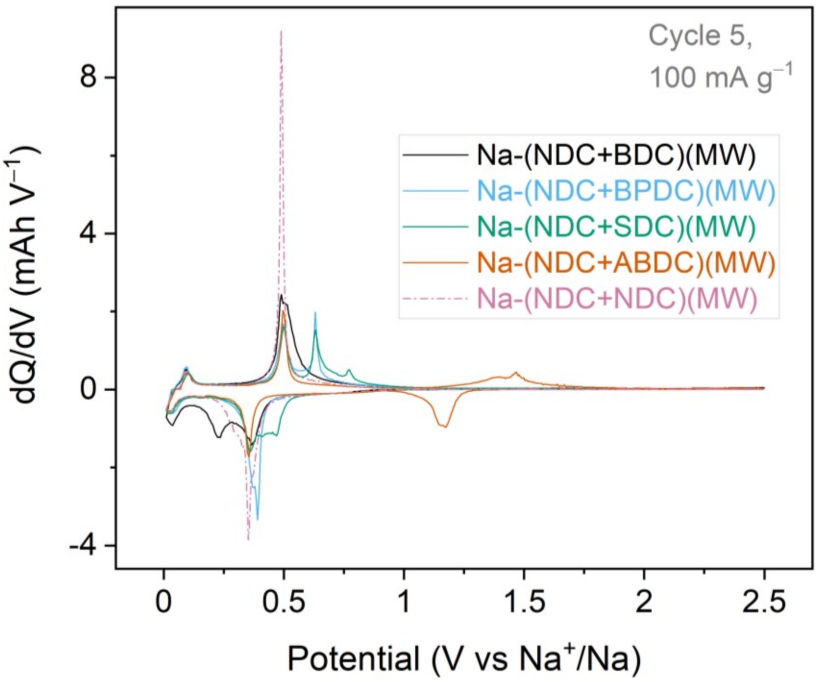

Figure S24. Differential capacity plot for the 5th cycle for all mixtures when cycled at 100 mA g^{-1}, compared to Na-(NDC+NDC)(MW).

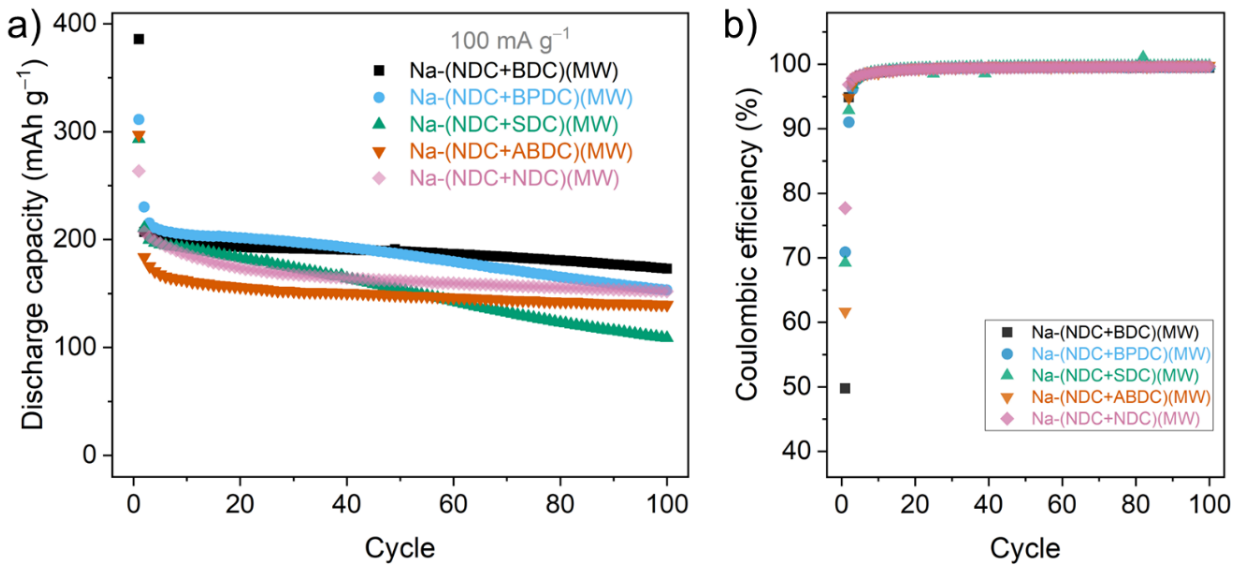

Figure S25. a) Discharge capacities and b) Coulombic efficiencies over 100 cycles for all the mixtures and Na-(NDC+NDC)(MW), cycled between 0.01-2.5 V at 100 mA g^{-1}.

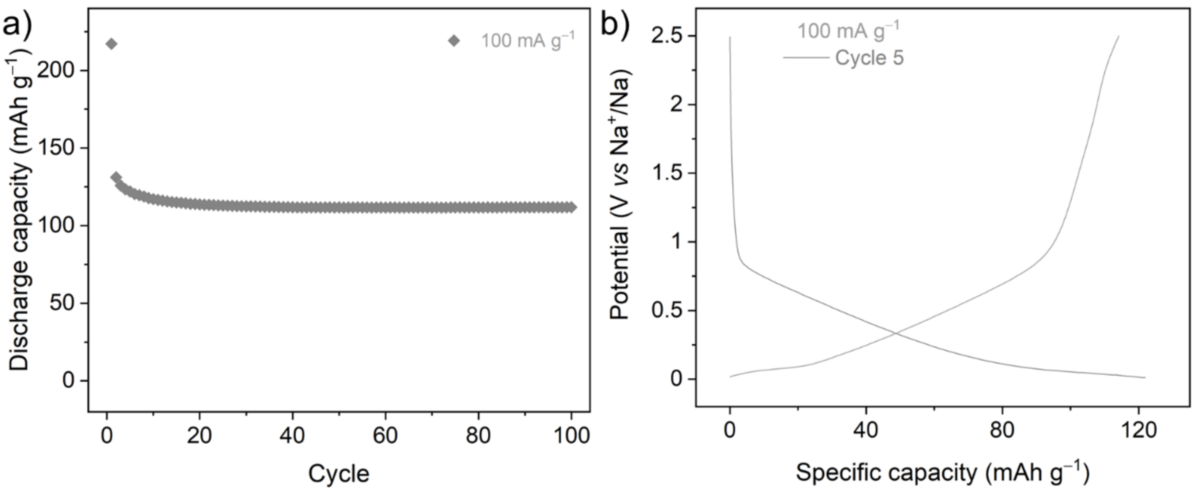

Figure S26. a) Discharge capacities over 100 cycles and b) voltage curve for the 5th cycle for an electrode containing only the carbon (90%) and binder (10%), cycled in half cell between 0.01-2.5 V at 100 mA g^{-1}. Based on the stable capacity of 112 mAh g^{-1}, maximum capacity contribution to electrodes prepared using 30% carbon would be 37.33 mAh g^{-1}.

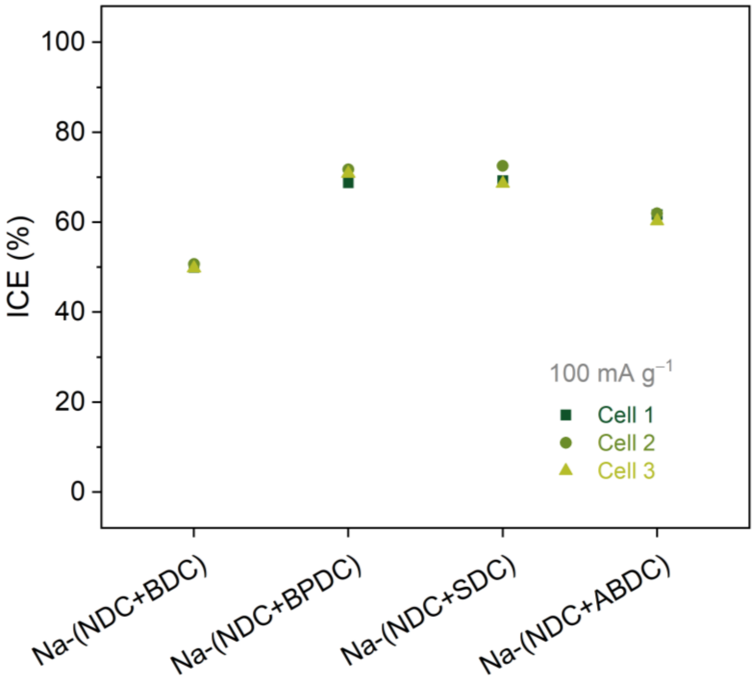

Figure S27. ICE values for 3 cells of all the mixtures cycled between 0.01-2.5 V at 100 mA g^{-1}.

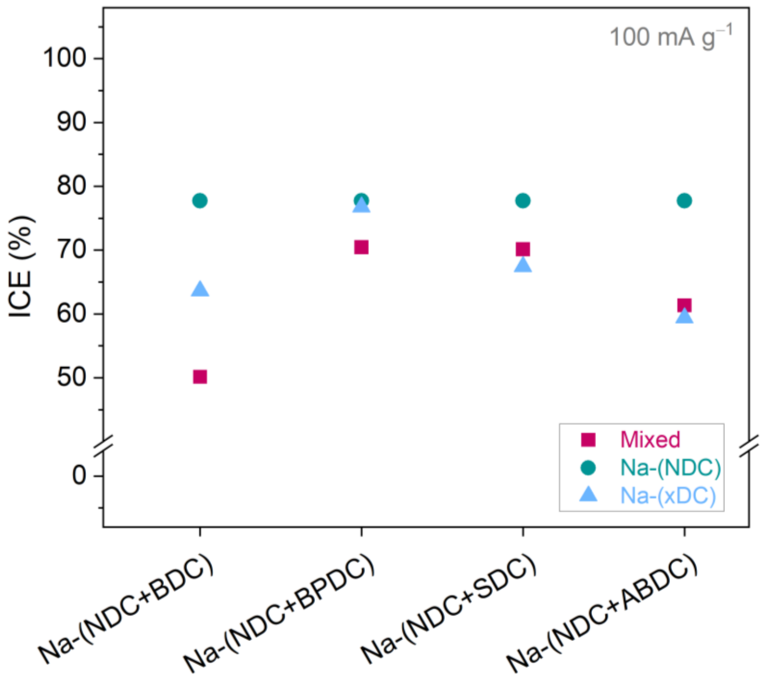

Figure S28. Comparison of ICE values for all the mixtures, relative to Na-(NDC)(MW) and respective individual sodium carboxylates [Na-(*x*DC)(MW)], cycled between 0.01-2.5 V at 100 mA g^{-1}. The numbers for mixed materials are an average value of 3 cells.

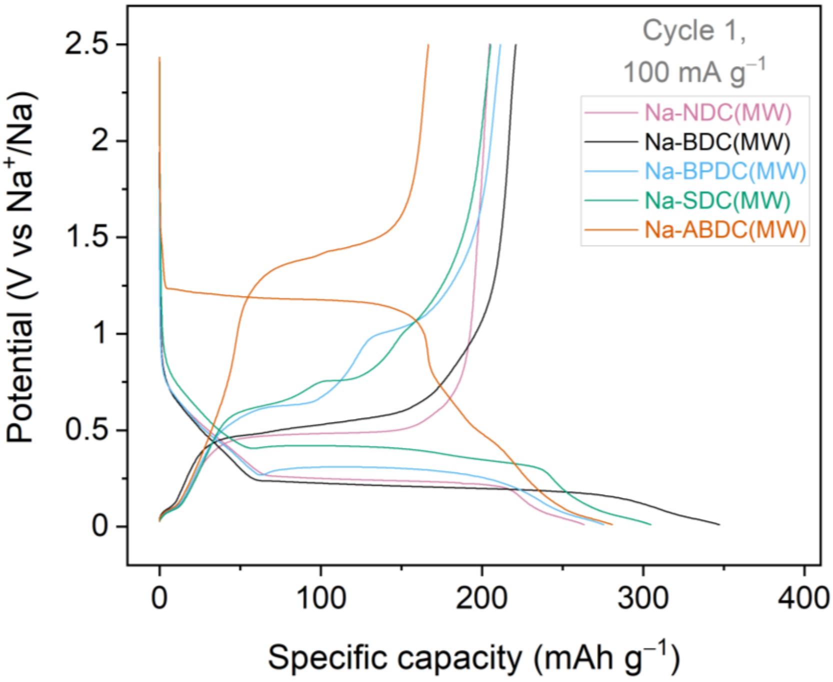

Figure S29. Charge/discharge traces for the first cycle of all the single component sodium carboxylates, cycled between 0.01-2.5 V at 100 mA g^{-1}.

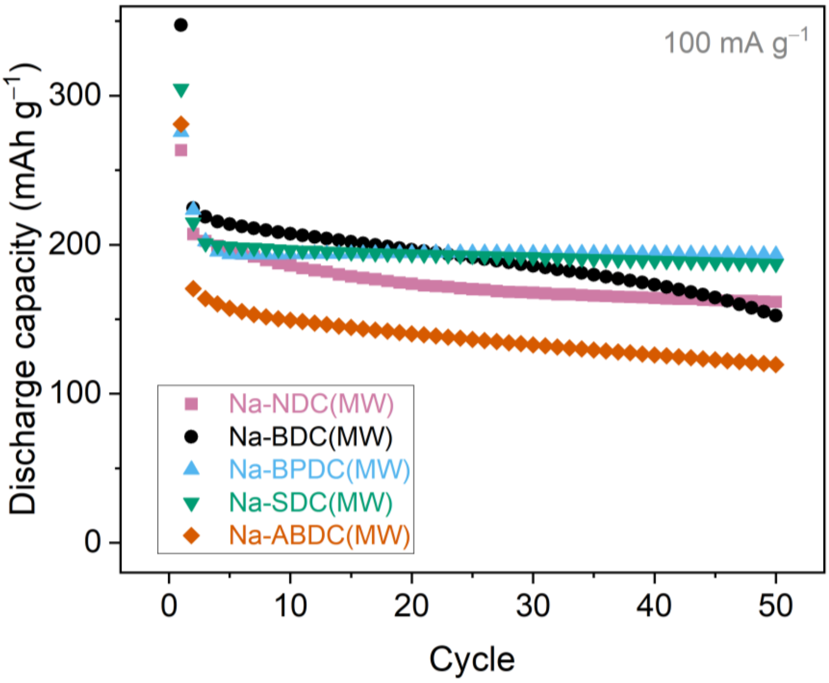

Figure S30. Discharge capacities for all the single component sodium carboxylates, cycled between 0.01-2.5 V at 100 mA g^{-1}.

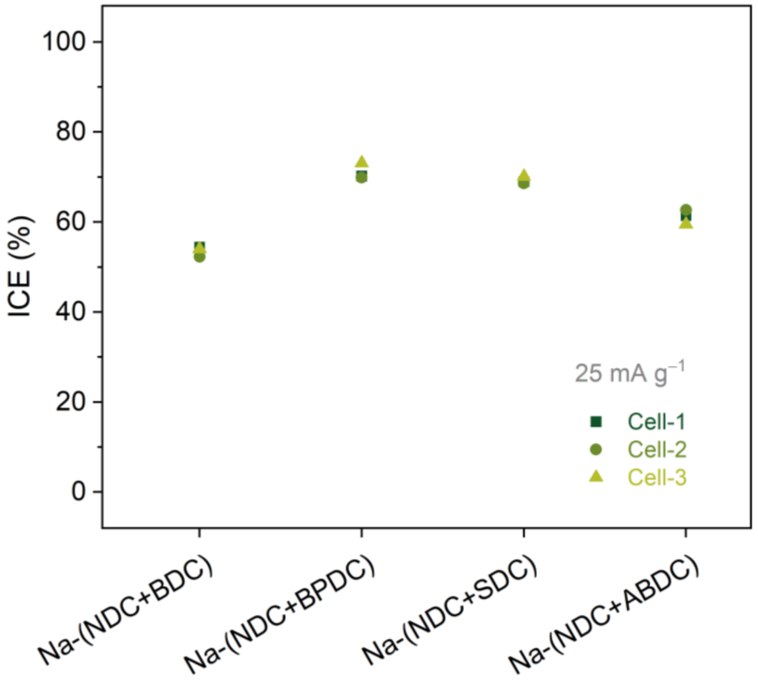

Figure S31. ICE values for 3 cells of all the mixtures cycled between 0.01-2.5 V at 25 mA g^{-1}.

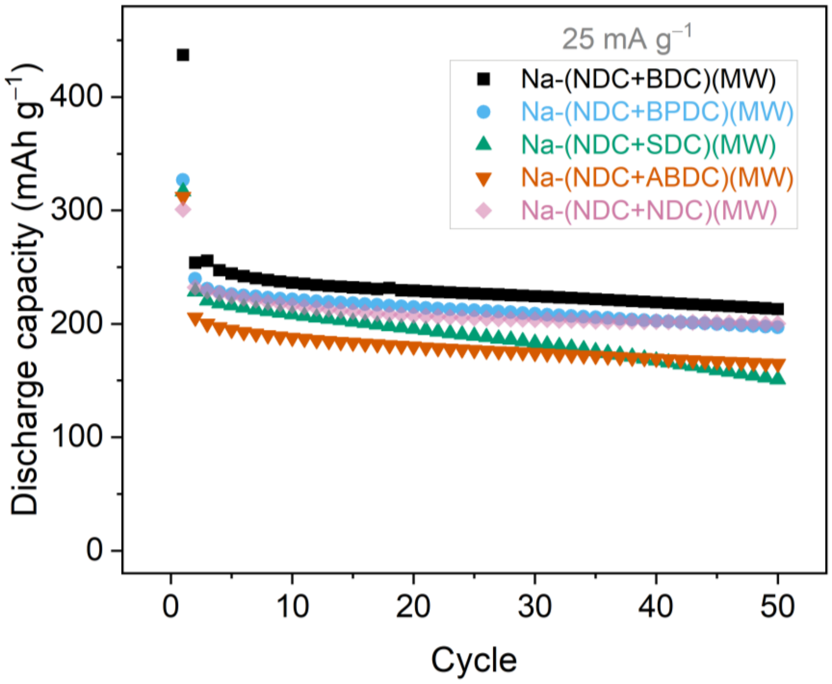

Figure S32. Discharge capacities for all the mixtures and Na-NDC(MW) upon galvanostatic cycling over 50 cycles at 25 mA g^{-1} between 0.01-2.5 V.

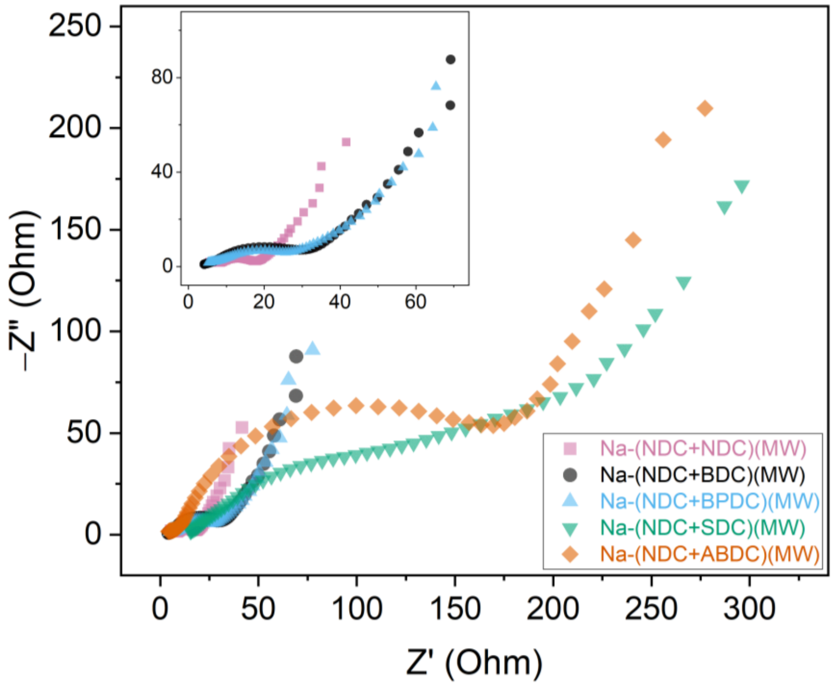

Figure S33. Nyquist plots for all materials obtained in symmetric cell format after completing one discharge-charge cycle. Inset: Zoomed version showing the plots for Na-(NDC+NDC)(MW), Na-(NDC+BDC)(MW) and Na-(NDC+BPDC)(MW).

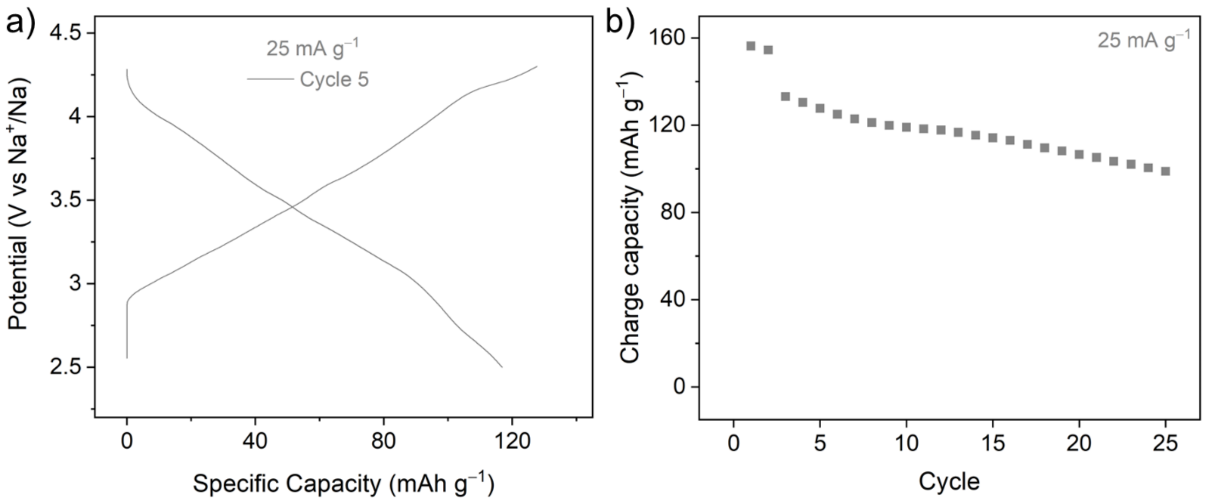

Figure S34. a) Voltage curve for the 5th cycle and b) discharge capacities over 25 cycles for the cathode material cycled in half cell between 2.5-4.3 V at 25 mA g^{-1}.

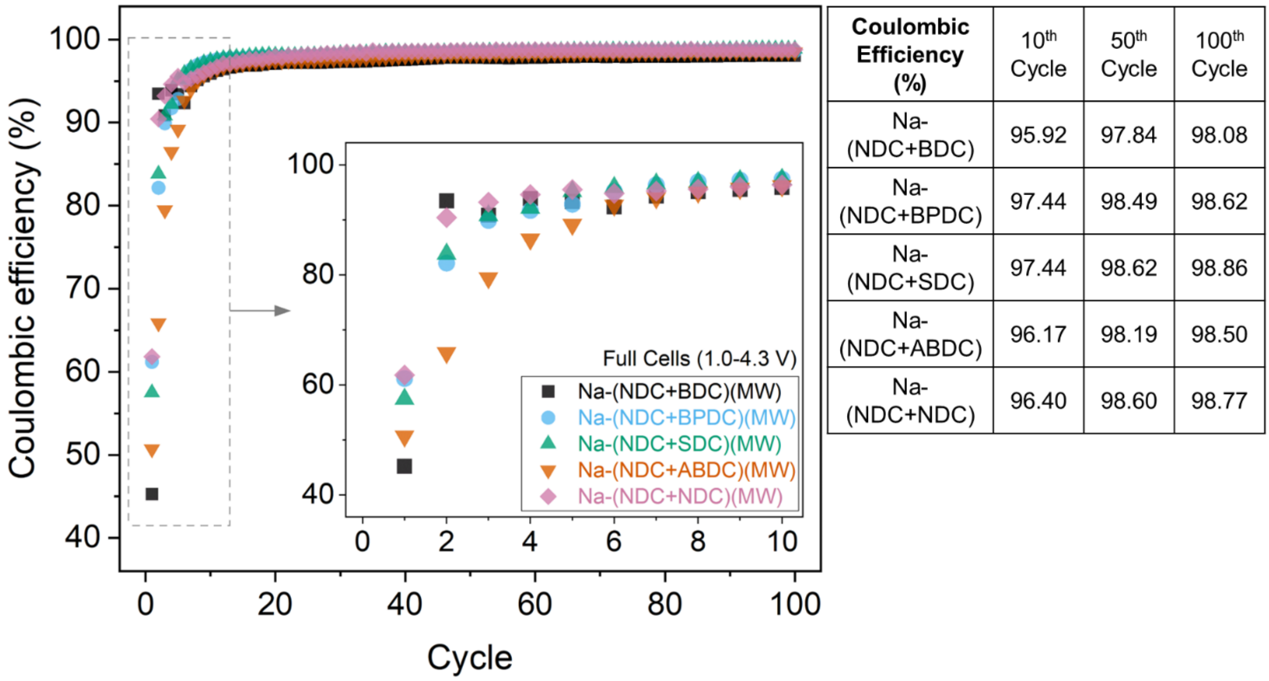

**Coulombic Efficiency (%)**	10th Cycle	50th Cycle	100th Cycle
Na-(NDC+BDC)	95.92	97.84	98.08
Na-(NDC+BPDC)	97.44	98.49	98.62
Na-(NDC+SDC)	97.44	98.62	98.86
Na-(NDC+ABDC)	96.17	98.19	98.50
Na-(NDC+NDC)	96.40	98.60	98.77

Figure S35. Coulombic efficiencies (CE) for full cells of binary mixtures and single component Na-NDC(MW). Inset: zoomed version showing the change in CE over the first 10 cycles. (Right) Table with the CE values for the 10th, 50th and 100th cycle.

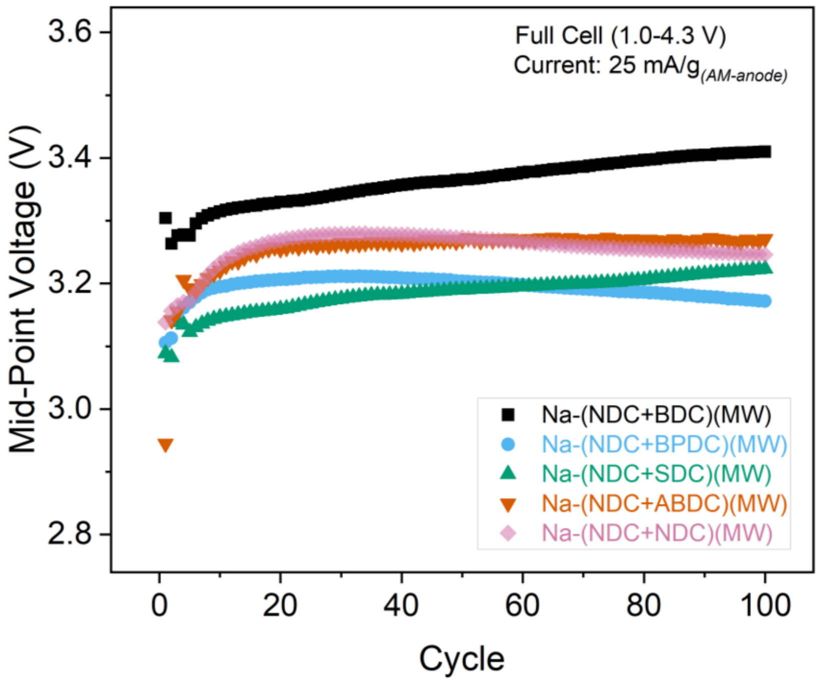

Figure S36. Mid-point voltage (MPV) values for full cells of the binary mixtures and single component Na-NDC(MW). MPV values have been computed as the voltage corresponding to 50% of the discharge capacity in every cycle.

Reference

1 T. Whewell, V. R. Seymour, K. Griffiths, N. R. Halcovitch, A. V Desai, R. E. Morris, A. R. Armstrong and J. M. Griffin, *Magn. Reson. Chem.*, 2022, **60**, 489–503.
